# Synthesis and characterization of heterometallic rings templated through alkylammonium or imidazolium cations†

**DOI:** 10.1039/d3dt00982c

**Published:** 2023-05-17

**Authors:** Rajeh Alotaibi, Amy Booth, Edmund Little, Adam Brookfield, Amritroop Achari, Selena J. Lockyer, Grigore A. Timco, George F. S. Whitehead, Iñigo J. Vitórica-Yrezábal, Nicholas F. Chilton, Rahul R. Nair, David Collison, Richard E. P. Winpenny

**Affiliations:** a Department of Chemistry and Photon Science Institute, The University of Manchester Oxford Road Manchester M13 9PL UK richard.winpenny@manchester.ac.uk; b Department of Chemical Engineering and Analytical Science and National Graphene Institute, The University of Manchester Oxford Road Manchester M13 9PL UK

## Abstract

We report the synthesis and structural characterization of a series of heterometallic rings templated *via* alkylammonium or imidazolium cations. The template and preference of each metal's coordination geometry can control the structure of heterometallic compounds, leading to octa-, nona-, deca-, dodeca-, and tetradeca-metallic rings. The compounds were characterized by single-crystal X-ray diffraction, elemental analysis, magnetometry, and EPR measurements. Magnetic measurements show that the exchange coupling between metal centres is antiferromagnetic. EPR spectroscopy shows that the spectra of {Cr_7_Zn} and {Cr_9_Zn} have *S* = 3/2 ground states, while the spectra of {Cr_12_Zn_2_} and {Cr_8_Zn} are consistent with *S* = 1 and 2 excited states. The EPR spectra of {(ImidH)-Cr_6_Zn_2_}, {(1-MeImH)-Cr_8_Zn_2_}, and {(1,2-diMeImH)-Cr_8_Zn_2_} include a combination of linkage isomers. The results on these related compounds allow us to examine the transferability of magnetic parameters between compounds.

## Abstract 2

Seven new heterometallic {Cr_*x*_Zn_*y*_} rings are reported, ranging in size from octametallic to tetradecametallic; their structures, magnetism and EPR spectra are reported.

## Graphical abstract

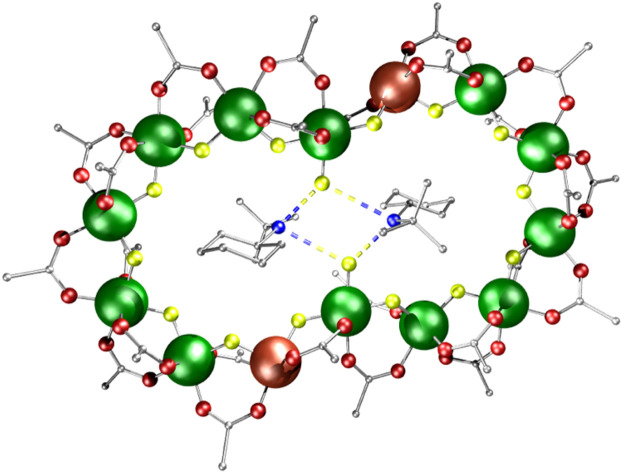

## Introduction

Template synthesis was one of the earliest methods used to synthesise macrocyclic ligands.^1^ Later, Raymond and others^2–6^ demonstrated that addition of templating counter-ions can be used to control the structure of polymetallic complexes, for example converting metal double-helices into metal tetrahedra. Particularly relevant to the present study is Pecoraro's work on metallocrowns,^3^ and Saalfrank's work on metallocoronates.^4^ We have reported the templating of heterometallic rings, using dialkylammonium cations such as di-*n*-propylammonium to form {Cr_7_M^II^} rings^7,8^ whereas the more sterically demanding cation di-i-propylammonium leads to {Cr_8_M^II^} rings.^9,10^ The templates have also included imidazolium cations, which can stack within the rings which then requires the ring to contain two M^II^ sites.^11^ When there are two heterometals within the ring the possibility of linkage isomerism arises. While linkage isomerism is taught to all undergraduate chemists,^12^ when discussing the binding of ligands such as thiocyanate^13^ or nitrite/nitrito,^14^ linkage isomerism due to variation in position of metals in heteropolymetallic complexes is rare.^15^

The family of heterometallic rings we have developed has been proposed as qubits for quantum computing,^16^ and as lithographic resists.^17^ We have also shown that the parent homometallic ring {Cr_8_} can act as a metallocrown towards CO_2_,^18^ and have bound other cations such as caesium to the centre of heterometallic rings.^19^ There is a danger that as we seek applications of these attractive molecules we might fail to understand the synthesis and structural chemistry. Therefore, here we are interested in the range of compounds that can be made and, specifically the transferability of structural features and magnetic parameters between different heterometallic rings but containing the same basic building blocks. The results also demonstrate the challenges that can occur when isomeric polymetallic compounds are found from a synthetic strategy. Here we describe {Cr_*x*_Zn_*y*_} rings (*x* = 6, 7, 8, 9, 12; *y* = 1 or 2) templated by bulky-alkylammonium or imidazolium cations. In addition to the synthesis and structural studies we describe magnetic and EPR spectroscopic investigations that demonstrate linkage isomerism is found in some cases.

## Results

### Synthesis and structures

All compounds were prepared by reacting chromium trifluoride hydrate, 2ZnCO_3_·3Zn(OH)_2_, and pivalic acid at 140–160 °C and a saturated amine or imidazole, which acts as a template once protonated. The new compounds to be discussed and yields are given in Table 1. Crystal data and refinement parameters are given in Table S1.†^20,21^ The compounds were obtained by crystallization and extraction to separate them from the homometallic ring [CrF(O_2_C^*t*^Bu)_2_] 1 which forms as a byproduct. Crystals could be grown from a mixture of solvents such as THF/MeCN, Et_2_O/MeCN, toluene/MeCN or hot THF. Compounds 2, 3, 5, 6 and 7 were all obtained from a reaction carried out for 24 h, at 140 °C. For 4, there was a 4 h reaction time at 140 °C and an additional 2 h heating at 160 °C. For 8 the reaction was carried out at 140 °C for 72 h. Elemental analysis (C, H, N, Cr, Zn) is good in all cases, although there is a distinction between compounds 2–5 where the elemental analysis fits with the retention of lattice solvent (Table S1†), while in 6–8 the elemental analysis indicates loss of lattice solvent.

**Table 1:** New compounds reported with number of isomers, type of chain present and yield of crystalline material

	Formula	Linkage isomers	Chains	Yield^a^/%
2	[2,4-diMe-Imid] [Cr_7_ZnF_8_(O_2_C^*t*^Bu)_16_]	1	{Cr_7_}	33
3	[ImidH]_2_ [Cr_8_ZnF_11_(O_2_C^*t*^Bu)_17_]	1	{Cr_8_}	6
4	[Cy_2_NHMe] [Cr_9_ZnF_12_(O_2_C^*t*^Bu)_18_]	1	{Cr_9_}	20
5	[ImidH]_2_ [Cr_6_Zn_2_F_8_(O_2_C^*t*^Bu)_16_]	5A	{Cr_6_}	55
		5B	Cr and {Cr_5_}	
		5C	{Cr_2_} and {Cr_4_}	
		5D	2 × {Cr_3_}	
6	[1-MeImH]_2_ [Cr_8_Zn_2_F_12_(O_2_C^*t*^Bu)_18_]	6A	{Cr_3_} and {Cr_5_}	30
		6B	2 × {Cr_4_}	
7	[1,2-diMeImH]_2_ [Cr_8_Zn_2_F_12_(O_2_C^*t*^Bu)_18_]	7A	{Cr_3_} and {Cr_5_}	62
		7B	2 × {Cr_4_}	
8	[CyNH_2_^*t*^Bu]_2_ [Cr_12_Zn_2_F_16_(O_2_C^*t*^Bu)_26_]	1	2 × {Cr_6_}	18

a Yield is based on Cr and reported for sum of linkage isomers where appropriate.

While there are many possible ways to order the compounds, we have decided to discuss the rings containing a single Zn^II^ site before moving to the more complex cases where there are two Zn^II^ sites in the ring.

**Fig. 1:**
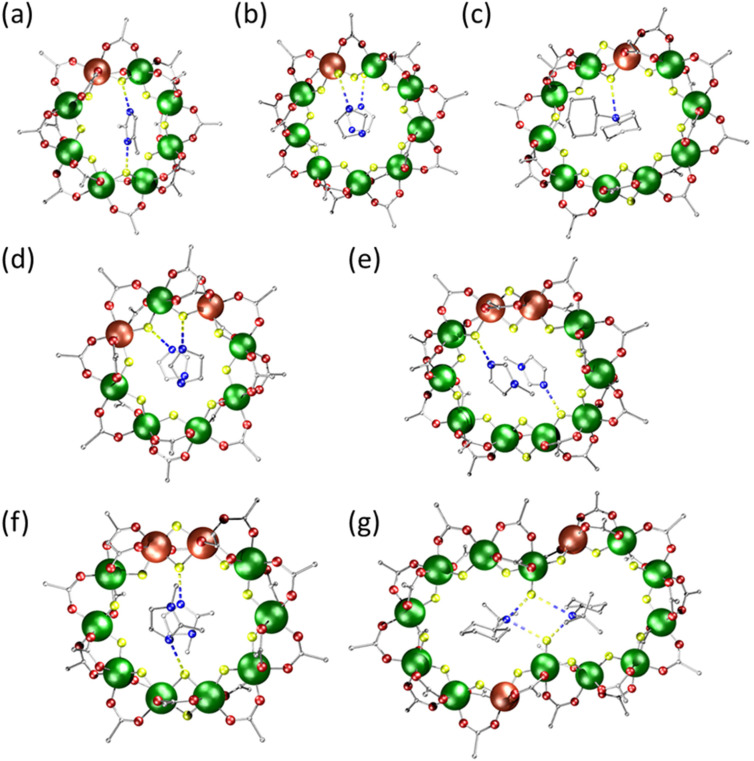
The structures of (a) 2; (b) 3; (c) 4; (d) 5; (e) 6; (f) 7; (g) 8. Cr dark green; Zn brown; O red; C grey; N dark blue; F yellow. The zinc sites are disordered as described in the text. N–H⋯.F hydrogen bonds shown as dashed lines. Hydrogen atoms and methyl groups of pivalate are omitted for clarity.

Using 2,4-dimethylimidazole (2,4-diMe-Imid) [2,4-diMe-ImidH][Cr_7_ZnF_8_(O_2_C^*t*^Bu)_16_] 2 formed (Fig. 1a). The structure is a regular octagon with one fluoride and two carboxylates on each edge: one carboxylate is in the plane of the metal octagon and one is perpendicular to the plane, alternating above and below the plane around the ring. The Zn^II^ site is disordered over the eight metal sites and a single dimethylimidazolium cation is found forming two N–H⋯F hydrogen bonds of 2.63 and 2.75 Å to bridging fluorides. This structure is similar to many other {Cr_7_M} rings.^7,8^

The compound [ImidH]_2_[Cr_8_ZnF_11_(O_2_C^*t*^Bu)_17_] 3 was synthesised as a minor product from a one-pot reaction using imidazole at 140 °C for 24 h; the major product was 5 (see below). Due to their different solubilities, they could be separated: 5 was extracted into hexane, whereas 3 was not soluble in hexane, and was extracted with hot THF. The enneametallic ring 3 contains eight metal sites bridged by one fluoride and two carboxylates (Fig. 1b), while the final bridge is formed by one fluoride and one carboxylate. Two metal centres contain a single terminal fluoride attached to the template through a H-bond in the final edges and the Zn(ii) is disordered between these two sites. Two cations are bound to the ring by two N–H⋯F hydrogen bonds of 2.66 Å. There are π-stacking interactions between the two cations with a centroid–centroid distance of 3.83 Å.

[Cy_2_NHMe][Cr_9_ZnF_12_(O_2_C^*t*^Bu)_18_] 4 was synthesized using a tertiary amine *N*-methyldicyclohexylamine (Cy_2_NMe). An oval-shaped 10-membered ring was formed (Fig. 1c). Eight edges are bridged by one fluoride and two pivalate groups, and two edges are bridged by two fluorides and one pivalate group. The Zn^II^ site is disordered between the four positions that are bridged by two fluorides. There is one-protonated ammonium inside the cavity of the ring to balance the charge. The protonated amine has a H-bonding interaction with a fluoride ion in one of the two edges bridged by a single carboxylate and two fluorides (N–H⋯F at 3.04 Å).

When 1,1-carbonyldiimidazole is used it decomposes during the reaction to give [ImidH]_2_[Cr_6_Zn_2_F_8_(O_2_C^*t*^Bu)_16_] 5 (where ImidH = imidazolium) (Fig. 1d); this is a cleaner route than starting from imidazole, which also gives 3. The structure is very similar to that of 2, but with two Zn^II^ disordered about the ring and two imidazolium cations at the centre of the ring with N–H⋯F hydrogen bonds of 2.84 to 2.91 Å. The imidazolium cations must have formed from reaction of the 1,1-carbonyldiimidazole added to the reaction. There are π-stacking interactions between the two cations, with a centroid–centroid distance of 4.10 Å.

1-methylimidazole (1-MeIm) and 1,2-dimethylimidazole (1,2-diMeIm) gave oval-shaped 10-membered rings with the formula [1-MeImH]_2_[Cr_8_Zn_2_F_12_(O_2_C^*t*^Bu)_18_] 6 and [1,2-diMeImH]_2_[Cr_8_Zn_2_F_12_(O_2_C^*t*^Bu)_18_] 7 (Fig. 1e and f, respectively). The structures of these two compounds are similar to that of 4. The Zn(ii) metal ions are disordered crystallographically over the four sites that are bridged by two fluorides. In 6, there are hydrogen bonds with N⋯F distances of 2.65 Å; in 7 there are hydrogen bonds with N⋯F distances of 2.68 Å. There are π-stacking interactions between the two cations in each case; in 6 the centroid–centroid distance is 3.44 Å, and in 7 the centroid–centroid distance is 3.45 Å.

[CyNH_2_^*t*^Bu]_2_[Cr_12_Zn_2_F_16_(O_2_C^*t*^Bu)_26_] 8 was prepared using *N-tert*-butylcyclohexylamine (CyNH^t^Bu) with a long reaction time (72 h, at 140 °C) and contains a 14-metal ring (Fig. 1g). Compound 8 has an hourglass shape that consists of two Zn(ii) and twelve Cr(iii) ions and is similar to a {Cr_10_Cu_2_} ring previously reported.^22^ The Zn(ii) atom sites are five-coordinate, and are linked by {Cr_6_} chains. There are two distinct Cr(iii) coordination sites, both six coordinate. Ten of the Cr(iii) sites are bound to two fluorides and four oxygen donors from carboxylates. The second Cr(iii) centre is located at the waist of the hourglass bound to a terminal fluoride, two bridging fluorides and three oxygens from bridging carboxylates. The Zn(ii) centres are bridged to the Cr(iii) centre at the waist by one fluoride and one carboxylate ligand and to a second Cr(iii) centre by one fluoride and two carboxylate ligands. All Cr⋯Cr edges are bridged by one fluoride and two carboxylate ligands. Two templates reside inside the ring's cavity through H-bonds with the terminal fluorides (N–H⋯F 2.68 Å). The two Zn(ii) atoms are crystallographically disordered over the four positions neighbouring the Cr(iii) centres at the waist of the hourglass.

### Magnetic studies

The magnetic susceptibility *χ*_M_ of compounds 2, 4, 5, 6, 7 and 8 was studied from 2–300 K. Compound 3 could not be isolated in sufficient quantity in a pure form to measure useful magnetic data. The plots of *χ*_M_*T*(*T*) for all compounds are similar and decrease with decreasing the temperature, which indicates the presence of antiferromagnetic interaction between metal centres. Fitting of the experimental data was completed using the program PHI^23^ through the spin Hamiltonian (eqn (1)).1
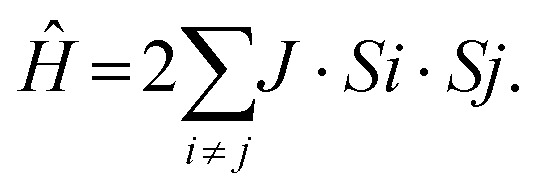


#### {Cr_*x*_Zn} heterometallic rings (*x* = 7 or 9)

**Fig. 2:**
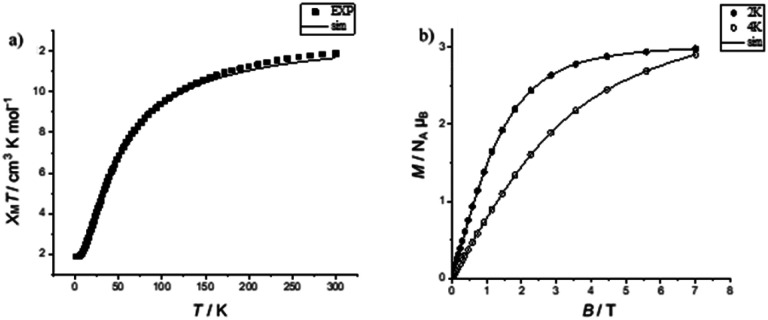
(a) Temperature-dependent *χ*_M_*T* of 2 in the temperature range 2 to 300 K, (measured at 1000 Oe); calculation for *J* = −5.2 cm^−1^, *g* = 1.99, the experimental data (■) and simulation (—). (b) Field-dependent magnetization of 2 at 2 (●) and 4 K (○); calculation for *J* = −5.2 cm^−1^, *g* = 1.99, and simulation (—).

In compound 2, the *χ*_M_*T* value is 11.89 cm^3^ K mol^−1^ at 300 K, which is below the expected value 12.99 cm^3^ K mol^−1^ for seven Cr(iii) ions, assuming *g* = 1.99. Upon cooling, the *χ*_M_*T* value is 1.85 cm^3^ K mol^−1^ at 2 K (Fig. 2a), which is close to the value of an isolated *S* = 3/2 state (1.80 cm^3^ K mol^−1^). Field-dependent magnetization measurements at 2 K show that the magnetization saturates at a value of 2.98 *N*_A_*μ*_B_ (2 K) at 7 T. This confirms an *S* = 3/2 ground state (Fig. 2b). The best fit values were for *J* = −5.2 cm^−1^, *g* = 1.99. These values were used when properties of linkage isomers were modelled (see below). This was done to reduce the number of parameters used in fitting the magnetic data of the more complicated compounds.

**Fig. 3:**
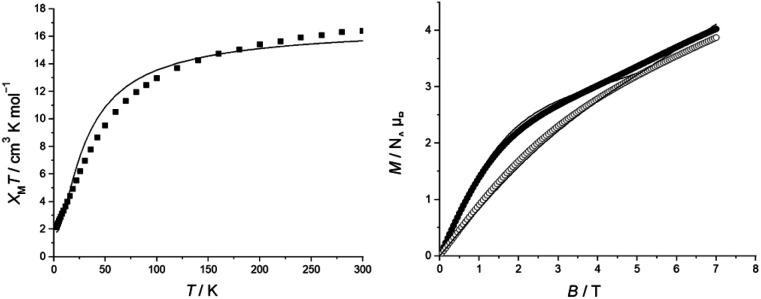
(a) Temperature-dependent *χ*_M_*T* of 4 in the temperature range 2 to 300 K (measured at 5000 Oe); the experimental data (■). (b) Field-dependent magnetization of 4 at 2 (●) and 4 K (○).

The *χ*_M_*T* value of compound 4 is 16.19 cm^3^ K mol^−1^ at 300 K, which is lower than the expected value 16.70 for nine isolated Cr(iii) ions (*g* = 1.99). Upon cooling, the *χ*_M_*T* value decreases to a minimum of 1.74 cm^3^ K mol^−1^ at 2 K (Fig. 3a). The magnetisation, *M*, at lower temperature (2 K) increases rapidly with increasing field, and it curves at a value of 3 *N*_A_*μ*_B_ by 3 T, which may suggest a ground state of *S* = 3/2 (Fig. 3b), but *M* continues to rise as B increases, also consistent with low lying excited states. Fitting *χ*_M_*T*(*T*) and *M*(B) simultaneously with PHI ^23^ gave *J* = −3.33(2) cm^−1^ and *g* = 1.998(3).

#### {Cr_*x*_Zn_2_} compounds (*x* = 6, 8, 12) and linkage isomerism

**Scheme 1:**
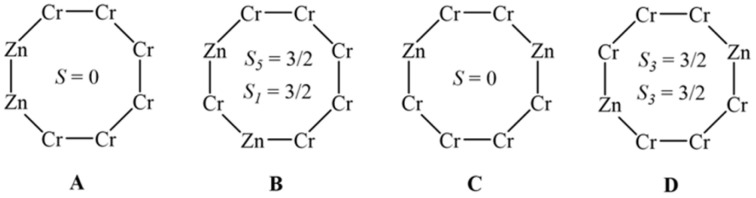
The four different possible isomers for 5. A and C would give a diamagnetic ground state, whereas B and D give a paramagnetic ground state.

In compound 5, there are six Cr(iii) ions and two Zn(ii) atoms. Crystallographically, the locations of zinc atoms are disordered over the ring, which leads to four possible isomers based on the zinc metal sites, whether they are on the odd or even sublattices. This gives two possible magnetic ground states shown in Scheme 1.

**Fig. 4:**
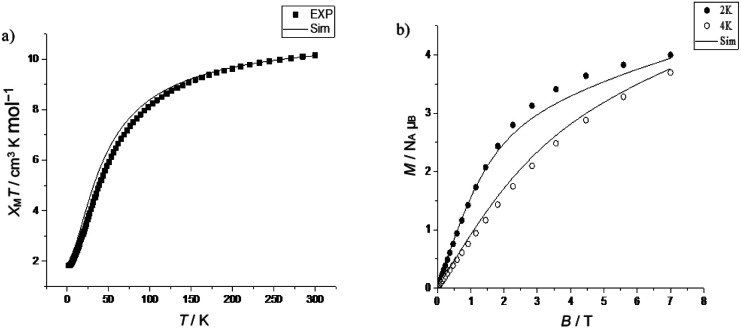
(a) Temperature-dependent χ_M_*T* of 5 in the temperature range 2 to 300 K, (measured at 1000 Oe); calculation for *J* = −5.2 cm^−1^, *g* = 1.99, the experimental data (■) and simulation (—). (b) Field-dependent magnetization of 5 at 2 (●) and 4 K (○); calculation for *J* = −5.2 cm^−1^, *g* = 1.99, the simulation (—, 2 and 4 K). The isomeric mixture is 50% of 5B and/or 5D (*S* = 3/2) and 50% of 5A and/or 5C (*S* = 0) ground state (see Scheme 1 for definition of isomers).

The *χ*_M_*T* value for 5 is 10.16 cm^3^ K mol^−1^ at 300 K, which is below the calculated value 11.13 cm^3^ K mol^−1^ for six Cr(iii) ions, and at 2 K is 1.83 cm^3^ K mol^−1^ (Fig. 4a). Field-dependent magnetization measurements at 2 K and 4 K show magnetization values of 4.00 *N*_A_*μ*_B_ (2 K) and 3.70 *N*_A_*μ*_B_ (4 K) at 7 T, and clearly the saturation of magnetization is not achieved, and there are probably low-lying excited state(s) (Fig. 4b).

In order to fit the magnetic data of compound 5, a statistical treatment of the possible isomers was used. Compound 5 includes four possible isomers: (Cr_6_ chain) isomer A, (Cr_5_/Cr_1_ chain) isomer B, (Cr_4_/Cr_2_ chains) isomer C, and (Cr_3_/Cr_3_ chains) isomer D (Scheme 1). Each chain was simulated through PHI ^23^ software using *g* = 1.99 and *J* = –5.2 cm^−1^ to gain the *χ*_M_*T* (T) and *M*(*H*) values. We use these values as they are those found for the octametallic ring 2. The values of these chains were combined together, giving the desired isomer (*e.g.* Cr_4_ chain + Cr_2_ chain = isomer C). The fitting began using this calculation: [(isomer A × 0.25) + (isomer B × 0.25) + (isomer C × 0.25) + (isomer D × 0.25)], meaning that 25% of each isomer is present, and this provided the best agreement in modelling the magnetic data. Varying the proportions of isomers A–D away from 25% of each led to a poorer fit to the data, and based on a Giant Spin Approximation,^24^ this gives a mixture of 50% diamagnetic (*S* = 0, A and C) and 50% paramagnetic (*S* = 3/2, B and D) ground states. It is impossible to differentiate between A and C, or B and D respectively from magnetometry alone. The EPR spectroscopy is a more informative guide here and shows the paramagnetic ground state must be from isomer D (see below).

**Scheme 2:**
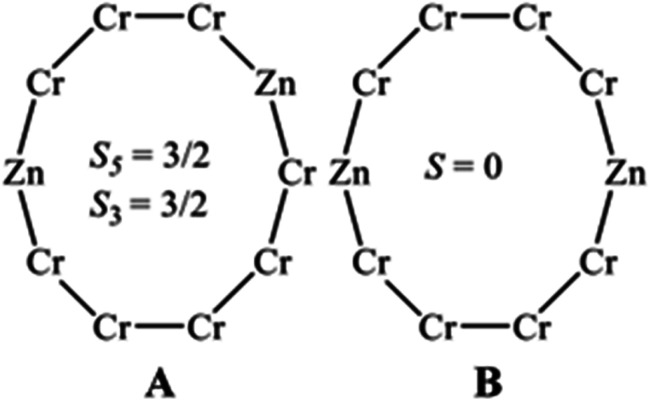
The two possible isomers for 6 and 7.

In compounds 6 and 7, there are eight Cr(iii) and two Zn(ii) ions. The two Zn^II^ sites are in the edge at the centre of the oval (Fig. 2c) and, assuming they do not neighbour each other, this gives only two possible isomers: A, which contains two odd-numbered chromium chains, both of which would have an *S* = 3/2 ground state; and isomer B, which would contain two four-chromium chains, which would have a diamagnetic ground state (Scheme 2). If the Zn^II^ sites were in the same edge this would give an eight-chromium chain, which would have a diamagnetic ground state.

**Fig. 5:**
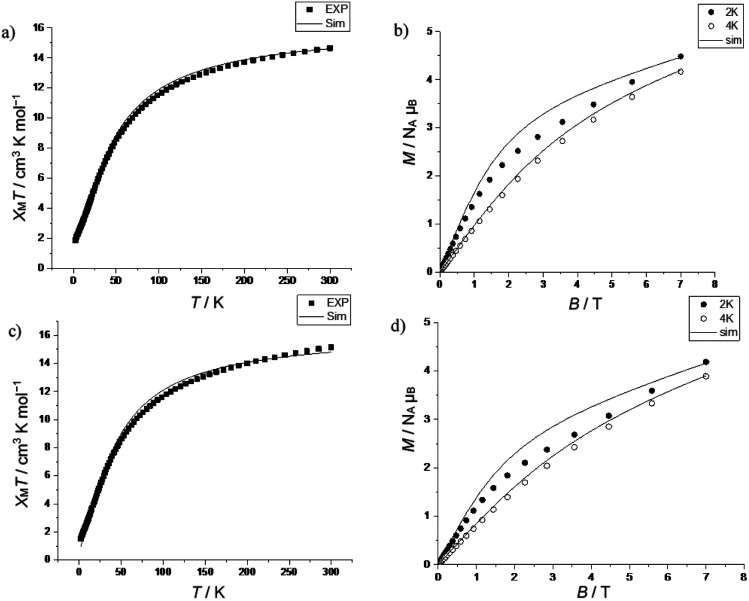
(a) and (c) Temperature-dependent *χ*_M_*T* of 6 and 7 in the temperature range 2 to 300 K, (measured at 1000 Oe); calculation for *J* = −5.2 cm^−1^, *g* = 1.99, the experimental data (■) and simulation (—). (b) and (d) Field-dependent magnetization of 6 and 7 at 2 (●) and 4 K (○); calculation for *J* = −5.2 cm^−1^, *g* = 1.99, the simulation (—, 2 and 4 K). The isomeric mixture for 6 is 50% : 50% 6A : 6B while for 7 it is 60% : 40% 7A : 7B. See Scheme 2 for definition of isomers.

The *χ*_M_*T* value for 6 is 14.65 cm^3^ K mol^−1^ at 300 K, which is slightly below the expected value 14.85 cm^3^ K mol^−1^ for eight Cr(iii) ions (*g*_Cr_ = 1.99) and at 2 K is 1.85 cm^3^ K mol^−1^, which is close to the value of an *S* = 3/2 state (1.80 cm^3^ K mol^−1^) (Fig. 5a). While for 7 the *χ*_M_*T* value at 300 K is 15.15 cm^3^ K mol^−1^, which is slightly above the expected value, and at 2 K is 1.51 cm^3^ K mol^−1^ (Fig. 5c). Field-dependent magnetization measurements of 6 and 7 at 2 K and 4 K show magnetization values of 4.48, 4.19 *N*_A_*μ*_B_ (2 K) and 4.17, 3.90 *N*_A_*μ*_B_ (4 K), respectively at 7 T (Fig. 5b and d). In both compounds, the magnetization does not saturate, which indicates excited states are being populated.

The data were fitted using PHI,^23^ with *J* = −5.2 cm^−1^ and *g* = 1.99, with only the relative mole ratios of the two isomers allowed to vary. In compound 6, the best agreement was achieved with 50% of isomer A and 50% of isomer B, while for compound 7 it is 60% of isomer A and 40% of isomer B. The fit of *χ*_M_*T*(*T*) is noticeably better than the fit of *M*(*B*), which is expected given the energy scales. A better fit may be possible by including inter-chain interactions or zero-field splitting of chromium(iii), but given the approximations being made this appears over-parameterization.

**Fig. 6:**
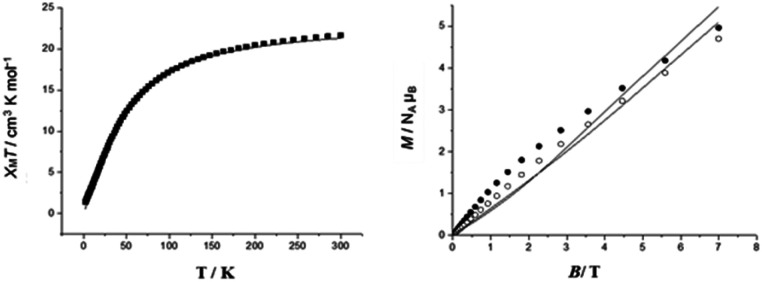
(a) Temperature-dependent *χ*_M_*T* of 8 in the temperature range 2 to 300 K (measured at 1000 Oe); the experimental data (■). (b) Field-dependent magnetization of 8 at 2 (●) and 4 K (○). Calculations for *J* = −5.2 cm^−1^, *g* = 2.05 shown as full lines.

Compound 8 contains two Zn(ii) metal centres which break the interaction between Cr(iii) ions, leaving two chains of {Cr_6_}. The *χ*_M_*T* value is 21.65 cm^3^ K mol^−1^ at room-temperature, which is very similar to the calculated value 21.61 cm^3^ K mol^−1^ for twelve non-interacting Cr(iii) ions (*g* = 1.96). At low temperature (2 K), the *χ*_M_*T* value falls to reach the value 1.36 cm^3^ K mol^−1^ (Fig. 6a). The magnetization (*M*) at 2 and 4 K is still increasing at the maximum accessible field of 7 T, which probably indicates low lying excited states are present (Fig. 6b).

The data were simulated as two {Cr_6_} chains, with a reasonable fit of *χ*_M_*T*(T) with *J* = −5.2 cm^−1^, *g* = 2.05; this *g*-value appears high for a chromium(iii) chain but the data did not fit as well for *g* = 1.99 (Fig. S1†). The fit of *M*(*B*) is poor, with 8 magnetising more quickly than would be predicted for a {Cr_6_} chain, which has an *S* = 0 ground state. The X-ray structure would allow the presence of an isomer containing {Cr_5_} and {Cr_7_} chains, which would fit the magnetisation behaviour, however, there are no signs of half-integer spin states in the EPR spectra of 8 (see below) and therefore we have not explored this further.

### EPR spectroscopy

**Fig. 7:**
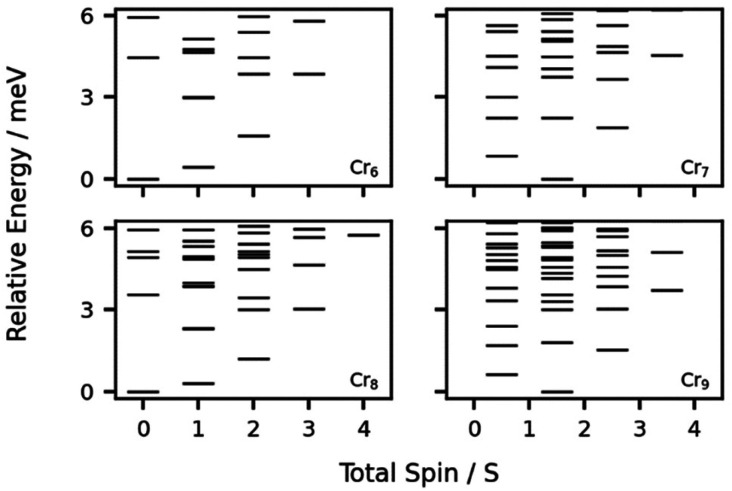
The calculated relative energies of the lowest lying spin states of antiferromagnetically coupled chains of chromium ions, using exchange values determined by inelastic neutron scattering.^25^ Further details of the calculations are included in the ESI.†

The EPR spectra were simulated using the Giant Spin Approximation, which is valid where the energy separation between spin states is significantly larger than the energy of any other term in the spin Hamiltonian.^24^ Within this approximation (assuming that any exchange interaction across Zn(ii) units is negligible) the compounds 8, 2, 3 and 4 represent, to a first approximation, antiferromagnetically coupled chains containing six, seven, eight, and nine Cr^III^ ions, respectively. For these compounds, the relative energies of the lowest lying spin states (Fig. 7), were calculated using *J* = −5.9 cm^−1^, as determined by inelastic neutron scattering (INS).^25^ The fit of the magnetic data for 4 suggest a smaller anti-ferromagnetic exchange interaction; this would bring the excited states closer in energy to the ground state and does not materially affect the discussion below.

**Fig. 8:**
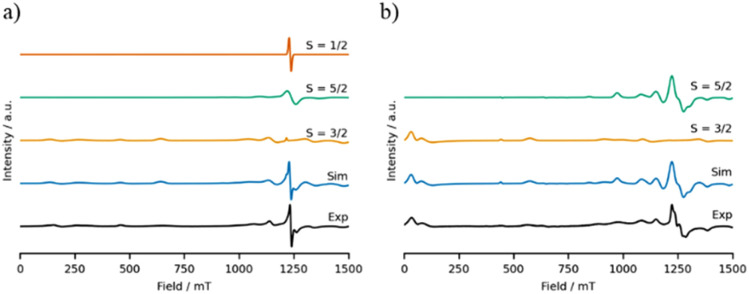
The EPR spectra (black) and simulations (blue) of polycrystalline samples of (a) 2 and (b) 4 at Q-band (34 GHz) at 5 K. The individual components of the simulations are shown stacked above the complete simulation, and the parameters used for these simulations are listed in Table 2.

**Fig. 9:**
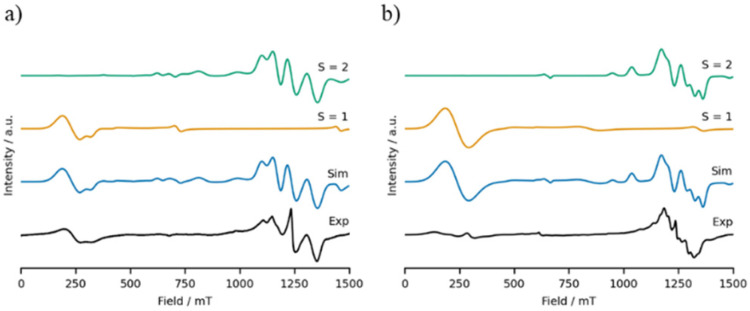
The EPR spectra (black) and simulations (blue) of polycrystalline samples of (a) 8 and (b) 3 at Q-band (34 GHz) at 5 K. The individual components of the simulations are shown stacked above the complete simulation, and the parameters used for these simulations are listed in Table 2.

Compounds 2 and 4 contain odd-numbered chromium chains, and so possess half-integer coupled spin states. The EPR spectra of these compounds are rich, with resonances across the field range at both Q- and K-bands (Fig. 8, S2–S4†) as has been observed previously for similar compounds.^26,27^ The features occurring below 750 mT at Q-band can be well simulated using Easyspin^29^ by considering the *S* = 3/2 ground state of these molecules. However to reproduce the resonances occurring in the region surrounding *g* = 2 (∼1250 mT at Q-band) it is necessary to consider additional spin states, which are shown by variable temperature EPR spectroscopy to correspond to thermal population of low-lying *S* = 1/2 and *S* = 5/2 excited states (Fig. S4†).^24^ Resonances arising from the excited states appear to dominate the EPR spectra, despite their low thermal population, as the excited state resonances occur in a narrow field range and are much sharper than those of the ground state, which has resonances across the entire field range. By contrast, compounds 8 and 3 contain even-numbered chromium chains, giving rise to integer coupled spin states. Since the ground state is diamagnetic, the EPR spectra of these molecules relate to the excited states (Fig. 9, S5–S7†).^26^ The EPR spectra of compounds 8 and 3 are as rich as those of compounds 2 and 4, and at Q-band give rise to broad resonances below 500 mT along with further resonances between 1100 mT and 1350 mT, consistent with previous reports of similar non-Kramers heterometallic clusters.^28^ The low field features can be reproduced using an *S* = 1 spin system, corresponding to the first excited state, while the resonances surrounding *g* = 2 arise due to thermal population of higher excited states (Fig. 9 and S7†), and can be simulated reasonably using an *S* = 2 spin system, consistent with previous findings for discrete even-numbered chains of antiferromagnetically coupled Cr(iii) ions.^26^ The axial zero field splitting parameter of the first excited states is much larger than those of the second excited states, being 5.6 and 9.5 times larger in compound 8 and 3, respectively, consistent with Kremer's observations in a prototypical Cr(iii) dimetallic bridged by three hydroxyl ions.^28^ However extraction of the single-ion axial zero field splitting and anisotropic exchange interactions is not trivial due to the extended length of the chromium chains,^27^ and consistent with what we have previously seen for antiferromagnetically coupled rings and chains.

**Table 2:** Magnetic parameters used in EPR simulations. All simulations were performed in the Matlab toolbox Easyspin^29^

Compound	Spin	Weight Q-band^a^	Weight K-band^a^	*g*	*D* (strain)/cm^−1^	*E* (strain)/cm^−1^	*H* Strain/cm^−1^
2	3/2	0.88	0.94	1.97	0.407 (0.061)	0.071 (0.014)	—
	1/2	0.07	0.06	1.97	—	—	0.010
	5/2	0.05	0.00	1.97	0.072	0.016	0.033
3	1	0.80	1	1.97	0.884 (0.088)	0.159 (0.0159)	—
	2	0.20	0	1.94	0.093	0.014	0.028
4	3/2	0.77	0.95	1.96	0.505 (0.025)	0.096 (0.024)	—
	5/2^b^	0.23	0.05	1.96	0.097	0.011	0.025
8	1	0.56	0	1.97	0.931	0.102	[0.020 0.067]^d^
	2^c^	0.44	1	1.98	0.167 (0.033)	0.029	[0.033 0.050]^d^

a Relative weights of spin state in simulation, Q-band at 5 K, K-band at 2.5 K.

b Simulation also includes *B*^0^_4_ = −1.0 and *B*^4^_4_ = 6.0 × 10^−14^ cm^−1.^

c Simulation also includes *B*^0^_4_ = 4.7 and *B*^4^_4_ = 1.7 × 10^−14^ cm^−1.^

d Indicates axial parameters, with the first and second values corresponding to the perpendicular (*x*,*y*) and parallel (*z*) regions, respectively.

The Zn(ii) ions present in compounds 5, 6, and 7 cannot be localised by X-ray crystallography, and so these compounds could consist of a combination of linkage isomers, as listed in Table 1 and outlined in Schemes 1 and 2 above.

**Fig. 10:**
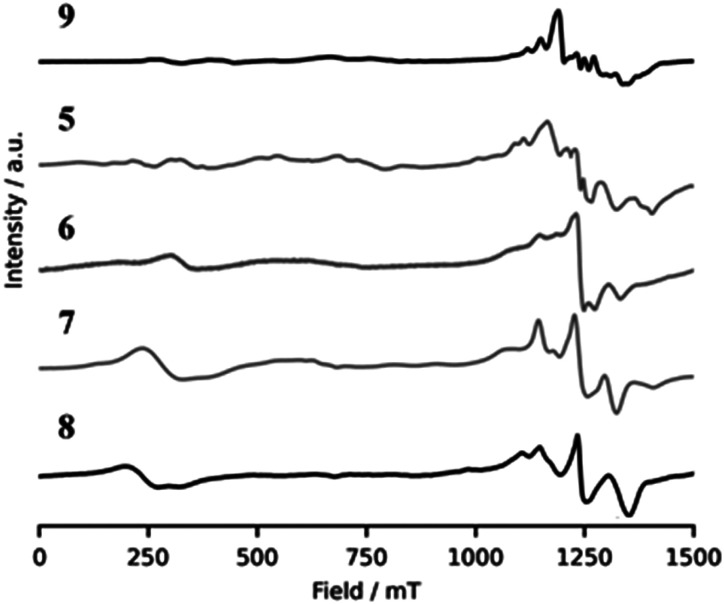
Comparison of the polycrystalline EPR spectra of 5–9 at Q-band at 5 K.

Compound 5 could contain any of four linkage isomers: for the isomers with *S* = 0 ground states (5A and 5C) we would expect to see spectra due to an *S* = 1 and possibly *S* = 2 excited states. For the isomers 5B and 5D we would expect to see the *S* = 3/2 ground state and *S* = 1/2 excited state; it is possible the *S* = 5/2 excited state contributes. Therefore, we could see contributions from at least six and possibly as many as ten spin states. The spectra of 5 are correspondingly rich (Fig. 10, S8–S10†), with resonances across the whole available field range. The spectra have some features reminiscent of 8, which contains two {Cr_6_} chains and therefore these features could be due to isomer 5A. Other features resemble those seen in an ordered {Cr_6_Zn_2_} structure [Me_4_N]_2_[Cr_6_M_2_F_8_(O_2_C^*t*^Bu)_16_] 9;^30^ these features would therefore be due to isomer 5D. Our conclusion is that both these isomers are present, but we cannot exclude the possibility that 5C is present; 5B would include features from a single Cr^III^ centre,^31^ and such features are clearly absent. We can therefore exclude 5B as a possible isomer. We have not attempted to simulate the spectra of 5, given their complexity.

We can apply similar analysis to the EPR spectra of compounds 6 and 7 (Fig. 10, S9 and S10†). There is a similarity between the Q-band spectra of compounds 6 and 7 with those of compound 8, which contains {Cr_6_} chains, and therefore our first thought is that isomers 6B and 7B (Scheme 2) are clearly present, *i.e.* those with even-number chromium chain.

There is less immediate evidence for the isomer containing odd-numbered chains, however the ground states of *S* = 3/2 for an odd-numbered chromium chain tends to have broad features and spread over the entire spectral window (see Fig. 7 above for {Cr_7_} and {Cr_9_} chains) and here we would have both {Cr_3_} and {Cr_5_} chains present, which would have similar but subtly different spin Hamiltonian parameters. The magnetic measurements indicate that isomers 6A and 7A, containing odd-number chains, are present in their respective samples, but there is no evidence from EPR spectroscopy.

Given the complexity of the spectra and the multiple isomers and spin states that may be present no attempt was made to simulate the spectra of compounds 6 or 7.

## Discussion

The synthetic route used is based on that used to produce previous heterometallic rings. The presence of larger cations introduces more complexity than we find with, for example secondary ammonium cations such as di-*n*-propylammonium. The difference is probably due to the different comparative stabilities of the structures formed. For the {Cr_7_M^II^} rings with Pr_2_NH_2_^+^ cations there is no other structure of comparable stability with the regular eight-metal ring where every metal⋯metal edge is the same, whereas for larger cations the resulting larger rings can be regular or can have edges that are bridged by two fluorides and a carboxylate rather than one fluoride and two carboxylates. Use of imidazolium cations produces another complication, which is that the imidazoliums can π-stack within the cavity of the ring, producing a dication. This in turn leads to a metal ring that is di-anionic, which is achieved by including two divalent metal ions. This then leads to the possibility of linkage isomerism. The complexity and the possibility of multiple products lead to, in general, lower yields for the compounds reported here when compared with those formed with simpler secondary ammonium cations.^7,8^

The linkage isomers found make interpretation of magnetic and EPR data complicated. For compound 5 the crystallography allows four possible linkage isomers. While we can rule out the isomer that involves a single Cr^III^ site and a {Cr_5_} chain from EPR spectroscopy (isomer 5B), the other three isomers are possible. The magnetic study suggests close to a 50 : 50 mixture of isomers with diamagnetic : paramagnetic ground states. This suggests 50% of compound 5D with the other 50% being 5A and 5C.

For compounds 6 and 7 crystallography allows two possible isomers, and the magnetic studies suggest an approximately equal distribution in the product. This contrasts with a family of {Cr_6_M_2_} rings we reported with tetramethylammonium as the cation.^30^

The magnetic study of 4 is a rare example of an open nine-metal chain. The best fit magnetic parameters show *J* = −3.33 cm^−1^, which is significantly smaller than the chromium–chromium exchange interactions found in seven-metal chains but similar to that found for a regular {Cr_9_} ring (*J* = −2.7 cm^−1^ using the same Hamiltonian used here).^32^ Therefore, it appears the larger rings are giving a less anti-ferromagnetic exchange interaction. Often an explanation for changes in the exchange interaction is sought in the bridging angle at μ_2_-ligands; in this case fluoride. In 2, comprising eight mono-fluoro-bridged units, the Cr–F–Cr angles range from 120.75(6)° Å to 124.02(6)°, average 122.99(17)°. In 4 the eight mono-fluoro-bridged units have comparable Cr–F–Cr angles ranging from 119.09(17)° to 123.80(17)°, average 122.43(45)°. This cannot be the source of the difference between the exchange interaction needed for 2 and 4.

In contrast the two di-fluoro-bridged units in 4 are much less obtuse ranging from 96.69(13) to 102.67(13)°; this could be predicted to give a less anti-ferromagnetic exchange. The very simple Hamiltonian we have used, which allows the data to be fitted, averages all the exchange interactions. An explanation of the lower average *J* in 4 could be that we are averaging two different exchange interactions; the first of which occurs on the eight mono-fluoro bridged edges, and which is similar to that in 2, the second of which occurs on the two di-fluoro bridged edges, and this is less anti-ferromagnetic. We have not fitted the data this way as there is no statistical justification for so doing.

## Conclusions

A series of cyclic heterometallic compounds with different structures has been successfully synthesised and characterized. The size of compounds can be controlled through the choice of template, which includes bulky-alkylammonium or imidazolium cations.

Magnetic studies show antiferromagnetic interaction between all metal centres and where there are two zinc centres in the ring linkage isomers are found, as shown by magnetic studies. Once linkage isomerism is present interpretation of the magnetism becomes challenging; here we present an approach based on minimising the parameters used and fixing the exchange interaction and *g*-value from smaller rings without linkage isomers. Other approaches are possible but at present we believe the data available only allow us to use the parameters discussed here.

## Author contributions

RA and AB(ooth) did the synthetic work, supervised closely by GAT. The X-ray structures were solved and refined by IV-Y and GFSW. EPR spectra were collected by EL and SJL. Magnetic data were collected by AB(rookfield), AA and RRN, and fitted by NFC. DC and REPW oversaw the project and wrote the manuscript with major input from RA and others. REPW navigated his way around the increasingly cumbersome bureaucracy of publishing.

## Conflicts of interest

There are no conflicts to declare.

## Supplementary Material

DT-052-D3DT00982C-s001

DT-052-D3DT00982C-s002
