# Correction: Synthesis and characterization of heterometallic rings templated through alkylammonium or imidazolium cations

**DOI:** 10.1039/d3dt90159a

**Published:** 2023-10-10

**Authors:** Rajeh Alotaibi, Amy Booth, Edmund Little, Adam Brookfield, Amritroop Achari, Selena J. Lockyer, Grigore A. Timco, George F. S. Whitehead, Iñigo J. Vitórica-Yrezábal, Nicholas F. Chilton, Rahul R. Nair, David Collison, Richard E. P. Winpenny

**Affiliations:** a Department of Chemistry and Photon Science Institute, The University of Manchester Oxford Road Manchester M13 9PL UK richard.winpenny@manchester.ac.uk; b Department of Chemistry, College of Science, King Saud University Riyadh Saudi Arabia; c Department of Chemical Engineering and Analytical Science and National Graphene Institute, The University of Manchester Oxford Road Manchester M13 9PL UK

## Abstract

Correction for ‘Synthesis and characterization of heterometallic rings templated through alkylammonium or imidazolium cations’ by Rajeh Alotaibi *et al.*, *Dalton Trans.*, 2023, **52**, 7473–7481, https://doi.org/10.1039/D3DT00982C.

The authors regret the omission of one of the affiliations for one of the authors, Rajeh Alotaibi, from the original manuscript. The corrected list of authors and affiliations for this paper is as shown above.

The Royal Society of Chemistry apologises for these errors and any consequent inconvenience to authors and readers.

## Supplementary Material

