# Supplementary material for: Synthesis and characterization of heterometallic rings templated through alkylammonium or imidazolium cations
Source: Dalton Trans. 2023 May 17;52(22):7473–81. doi: 10.1039/d3dt00982c (PMC10242454; doi:10.1039/d3dt00982c)
Supplement: DT-052-D3DT00982C-s001 [file DT-052-D3DT00982C-s001.pdf]

Electronic Supplementary Material (ESI) for Dalton Transactions.
This journal is © The Royal Society of Chemistry 2023

Supplementary Information

Synthesis and Characterization of Heterometallic Rings Templated through Alkylammonium or Imidazolium Cations

Rajeh Alotaibi, Amy Booth, Edmund Little, Adam Brookfield, Amritroop Achari, Selena J. Lockyer, Grigore A. Timco, George F. S. Whitehead, Inigo Vitorica-yrezabal, Nicholas F. Chilton, Rahul R. Nair, David Collison, and Richard E. P. Winpenny

Contents

S1. SYNTHESIS 2

S2. CRYSTALLOGRAPHY 6

S3. MAGNETIC DATA 10

S4. EPR SPECTROSCOPY 12

S1. Synthesis

All reagents were obtained from Aldrich. The syntheses of complexes were carried out in Erlenmeyer Teflon® FEP flasks supplied by Fisher. $[Co_2(H_2O)(O_2C^tBu)_4(HO_2C^tBu)_4]$ and $[Ni_2(H_2O)(O_2C^tBu)_4(HO_2C^tBu)_4]$ were prepared as previously reported.[29,30] The calculated elemental analyses are based on the unit cell contents from the X-ray diffraction studies (see Table S1 below). Elemental analysis of the complexes was obtained by The University of Manchester microanalytical service. The elemental analysis for **2** – **5** match the unit cell contents from the X-ray structures; for **6** – **8** the elemental analysis matches for loss of the lattice solvent. In all cases attempts to record powder diffraction patterns gave little diffraction, indicating amorphous materials.

[2,4-DiMe-ImidH]$[Cr_7ZnF_8(O_2C^tBu)_{16}]$ 2: $CrF_3.4H_2O$ (3.0 g, 17 mmol), 2,4-dimethylimidazole (0.6 g, 6.2 mmol), $2ZnCO_3.3Zn(OH)_2$ (0.7 g, 1.2 mmol), and $^tBuCO_2H$ (15.0 g, 147 mmol) were heated while stirring at 140 ºC for 24 h. The flask was cooled to room temperature and acetone (30 ml) was added to the mixture causing precipitation and stirred for 12 h. The green product was collected by filtration, washed with a large quantity of acetone, and then dissolved in hexane (250 ml) at room temperature with stirring for 1 h. The solution was filtered, and the filtrate was collected and solvent was removed from the extraction under vacuum. Yield 1.8 g (33%, calculated from the available Cr). Elemental analysis (%): calculated for $C_{85}H_{153}Cr_7ZnF_8N_2O_{32}$: Cr 15.85, Zn 2.85, C 44.45, H 6.72, N 1.22; found: Cr 15.01, Zn 3.06, C 44.98, H 6.98, N 1.28. The product was crystallized from a THF/MeCN (3:1 v/v) mixture by slow evaporation of the solvents to give X-ray quality crystals in 2 days.

$[ImidH]_2[Cr_8ZnF_{11}(O_2C^tBu)_{17}]$ 3: $CrF_3.4H_2O$ (3.0 g, 17 mmol), imidazole (0.7 g, 10 mmol), $2ZnCO_3.3Zn(OH)_2$ (0.3 g, 0.5 mmol), and $^tBuCO_2H$ (12.0 g, 118 mmol) were heated while stirring at 140 ºC for 24 h. The flask was cooled to room temperature, and acetone (30

ml) was added to the mixture causing precipitation and stirred for 12 h. The crude green product was collected by filtration, washed with a large quantity of acetone, and dried in air.

The green product was then treated with hexane (3 x 50 ml) with stirring for 1 h at room temperature. The residue that did not dissolve in hexane was then dissolved in hot THF (70 ml) and stirred for 1 h. The solution was filtered, and the solvent was removed from the filtrate under vacuum giving crude **3**. Yield 0.3 g (6%, calculated from the available Cr). Elemental analysis (%): calculated for $C_{93}H_{163}Cr_8F_{11}N_2O_{34}Zn$: Cr 16.35, Zn 2.57, C 43.91, H 6.46, N 1.10; found: Cr 16.48, Zn 2.76, C 43.01, H 6.48, N 2.13. The product was crystallized using vapour diffusion of MeCN into a toluene solution of the product to give X-ray quality crystals in two days. EPR measurements were performed on crystalline material, but there was insufficient for magnetic studies.

The hexane washings (3×50 ml) were collected, filtered and the solvent removed from the filtrate under vacuum giving crude **5**; a cleaner synthesis for **5** is given below. Yield 1.6 g (24 %, calculated from the available Cr). Elemental analysis (%): calculated for $C_{89.5}H_{158}Cr_6F_8N_4O_{32}Zn_2$: Cr 13.02, Zn 5.46, C 44.84, H 6.64, N 2.34; found: Cr 14.10, Zn 4.16, C 43.69, H 6.59, N 2.10.

$[Cy_2NHMe)][Cr_9ZnF_{12}(O_2C^tBu)_{18}]$ 4: $2ZnCO_3.3Zn(OH)_2$ (0.5 g, 0.91 mmol), $^tBuCO_2H$ (15 g, 147 mmol), N-methyldicyclohexylamine (2.6 g, 6.1 mmol), and $CrF_3.4H_2O$ (5.0 g, 28 mmol) were heated at 140 ºC with stirring for 0.5 h then the temperature was raised to 160 ºC for 4 h. The mixture was cooled to room temperature, acetone (50 mL) was added causing precipitation, and the reaction mixture stirred overnight. The product was collected by filtration and washed with a large quantity of acetone. The product was extracted into hot hexane (350 mL), the solution filtered and the solvent removed *in vacuo*, giving the title product as a green solid. Yield 1.7 g (20%, calculated from the available Cr). Elemental analysis (%): calculated for $C_{107}H_{196}Cr_9F_{12}NO_{37}Zn$: Cr 16.42, Zn 2.29, C 45.09, H 6.93, N 0.49; found: Cr 16.44, Zn

2.36, C 45.18, H 6.90, N 0.53. The product was crystallized from a THF/MeCN (3:1 v/v) mixture by slow evaporation of the solvents and gave X-ray quality crystals in two days.

[imidH]$_2$[Cr$_6$Zn$_2$F$_8$(O$_2$C^{t}Bu)$_{16}$] 5: Compound **5** was synthesised by a similar procedure as compound **2**, but by using 1,1-carbonyldiimidazole (0.7 g, 4.3 mmol) instead of 2,4-dimethylimidazole. Yield 4.2 g (65%, calculated from the available Cr). Elemental analysis (%): calculated for $C_{89.5}H_{158}Cr_6F_8N_4O_{32}Zn_2$: Cr 13.02, Zn 5.46, C 44.84, H 6.64, N 2.34; found: Cr 13.16, Zn 5.19, C 44.38, H 6.77, N 2.29. The product was crystallized using vapour diffusion of MeCN into a toluene solution of the product to give X-ray quality crystals in 2 days.

[1-MeImiH]$_2$[Cr$_8$Zn$_2$F$_{12}$(O$_2$C^{t}Bu)$_{18}$] 6: Compound **6** was synthesised by a similar procedure as compound **2**, but by using 1-methylimidazole (0.90 g, 10.9 mmol) instead of 2,4-dimethylimidazole. Yield 1.7 g (30%, calculated from the available Cr). Elemental analysis (%): calculated for the desolvated **6** $C_{100}H_{180}Cr_8F_{12}N_4O_{36}Zn_2$: Cr 14.91, Zn 4.69, C 43.06, H 6.50, N 2.01; found: Cr 14.24, Zn 4.66, C 43.00, H 6.48, N 1.97. The product was crystallized using vapour diffusion of MeCN into a toluene solution of the product to give X-ray quality crystals in 2 days.

[1,2-diMeImH]$_2$[Cr$_8$Zn$_2$F$_{12}$(O$_2$C^{t}Bu)$_{18}$] 7: Compound **7** was synthesised by a similar procedure as compound **2**, but by using 1,2-dimethylimidazole (0.6 g, 6.2 mmol) instead of 2,4-dimethylimidazole. Yield 0.7 g (12%, calculated from the available Cr). The portion of the crude product that did not dissolve in hexane was dissolved in hot THF and stirred for 1 h. The product was filtered and the solvent was removed from the extraction under vacuum. Yield 2.9 g (50%, calculated from the available Cr). Elemental analysis (%): calculated for desolvated **7** $C_{100}H_{180}Cr_8F_{12}N_4O_{36}Zn_2$: Cr 14.91, Zn 4.69, C 43.06, H 6.50, N 2.01; found: Cr 14.24, Zn 4.66, C 43.00, H 6.48, N 1.97. The product was crystallized using vapour diffusion of MeCN into a toluene solution of the product to give X-ray quality crystals in 2 days.

$[CyNH_2{}^tBu]_2[Cr_{12}Zn_2F_{16}(O_2C^tBu)_{26}]$ 8: $2ZnCO_3.3Zn(OH)_2$ (0.35 g, 0.6 mmol), $^tBuCO_2H$ (25 g, 245 mmol), N-tert-butylcyclohexylamine (0.95 g, 6.1 mmol), and $CrF_3.4H_2O$ (3.0 g, 17 mmol) were heated at 140 ºC with stirring for 72 h. The mixture was cooled to room temperature, acetone (30 mL) was added causing precipitation, and the reaction stirred overnight. The product was collected by filtration and washed with acetone. The product was extracted into hexane (100 mL), filtered and the filtrate solvent was removed *in vacuo*, giving the title product as a green solid. Yield 1.0 g (18%, calculated from the available Cr). Elemental analysis (%): calculated for desolvated **8** $C_{150}H_{266}Cr_{12}F_{16}N_2O_{52}Zn_2$: Cr 15.64, Zn 3.28, C 45.17, H 6.72, N 0.70; found: Cr 15.50, Zn 2.87, C 45.18, H 7.04, N 0.59. The product was crystallized from hot THF and gave X-ray quality crystals in two days.

S2. Crystallography

Data collection: X-ray diffraction data were collected for compounds **2-7** on a dual source Rigaku FR-X rotating anode with Cu-Kα (1.54184 Å) or Mo-Kα (0.71073 Å) radiation (see Table S1), equipped with a Hypix-6000HE detector and Oxford Cryostream 800 plus cryosystem. The temperatures of the diffraction experiments are also given in Table S1. X-ray diffraction data for compound **8** were collected using a Rigaku Supernova X-ray diffractometer with a microfocus Mo- Kα (0.71073 Å) source, equipped with a Eos CCD detector and Oxford Cryostream 700 cryosystem. All data were collected using CrysAlisPro software.[1]

Crystal structure determination and refinements: X-ray data were processed and reduced using CrysAlisPro. Absorption correction was performed using empirical methods (SCALE3 ABSPACK) based upon symmetry-equivalent reflections combined with measurements at different azimuthal angles. The crystal structure was solved and refined against all F^2 values using the SHELX and Olex2 suite of programmes[2,3] All atoms were refined anisotropically. Hydrogen atoms were refined using the riding model using idealised geometries and riding isotropic displacement parameters based on the parent atom. Compounds **3** and **7** were disordered and modelled over two positions. All compounds had disordered pivalate ligands, cationic threads and solvent molecules that were modelled over two or more positions where possible. The 1,2- and 1,3- interatomic distance of equivalent disordered ~~pairs~~ moieties ~~of atoms~~ were restrained to be similar using same distance restraints. The atomic displacement parameters of the disordered moieties were also restrained using enhanced rigid bond and similar neighbouring atomic displacement parameter restraints. Data for crystal structures **3**, **4** and **8** were found to be twinned. Despite the use of a high intensity microfocused X-ray source, crystals of **8** only diffracted to 0.95 Å resolution. The data were truncated accordingly. The Zn/Cr relative atomic occupancies were refined in all crystal structures, except in structure 7, where the disordered difluoride bridging edge precluded such a refinement and the Zn/Cr ratio

was fixed at 1:1 for the disordered pairs. Sum of parts restraints were used to restrain the Zn/Cr occupancies to that expected for structure neutrality. ~~Once the Zn atomic positions were identified, the occupancies were set and t~~The atomic Zn/Cr atomic positions and displacement parameters were constrained to be equal using equal atomic displacement parameter and equal coordinate constraints. Where the Cr/Zn sites could be identified from the relative occupancies in the sum of parts restraint, the number of sites the Zn occupancy was refined against was reduced accordingly. In all cases, it was felt application of a solvent mask to model these disordered solvent molecules was not appropriate, as accompanying TGA or elemental analysis data could not be obtained to back up any conclusions drawn from the calculated contents of the solvent mask

Additional notes

2

The structure contains a solvent accessible voids. No appreciable electron density could be found in these voids; the voids are empty.

3

The data were found to be non-merohedrally twinned, with two clear reciprocal lattices observed rotated by 120° around [0 0 1]. Treating the data as non-merohedrally twinned eliminated a number of artifacts from the electron density difference map compared to data reduction as a single component. The whole molecule has been refined disordered over two positions across a 2-fold rotation operation. Reducing the symmetry of the model did not resolve this disorder, nor improve the outcome of the refinement.

4

PLATON CheckCIF calculates that this structure exhibits a large range of non-solvent Ueq's for carbon atoms in the structure. This is due to the methyl groups of the pivalate moieties

exhibit a larger apparent thermal motion than the neighbouring pivot atoms of the tbutyl moiety, indicative of statistical disorder of these groups within the crystal. This results in a large range of Ueq for C's within the structure

6

The whole molecule has been refined disordered over two positions across an inversion centre. Reducing the symmetry of the model did not resolve this disorder, nor improve the outcome of the refinement. The data have low completeness; The crystal slipped during the data collection. Luckily it was possible to integrate all data available, but the slip resulted in part of the unique data to not be recorded. Unfortunately, it was not possible to recollect an additional data-set as the issue was only discovered long after the data collection was made. PLATON CheckCIF calculates that this structure exhibits a large range of non-solvent Ueq's for carbon atoms in the structure. However, despite the assertions of PLATON, this measure has included the disordered and admittedly poorly localised solvent molecules (as can be seen from the moiety formula in the CheckCIF report for this structure, C121 H203 Cr8 F12 N5 O36 Zn2, rather than the reported 2(C4 H7 N2), 3(C7 H8), C90 H162 Cr8 F12 O36 Zn2, C2 H3 N; the solvent molecules have not been counted as separate in the moiety formula calculation). Refinement of the model with the solvent molecules removed suppresses this alert.

7

This structure shows evidence of rotational disorder of the whole Cr_8Zn_2 ring that could not be satisfactorily modelled. The difluoride bridging edge is disordered with an approximately 4:1 ratio with a "normal" equatorial bridging pivlate and single fluoride on the inner edge of the ring. This disordered group is accompanied on either side by small regions of electron density in the Fo-Fc difference map than can be attributed to low occupancy difluoride bridges (both ¼ the occupancy (or ~5% total occupancy) of the disordered "normal" edge, due to the resulting possible orientations of the ring about the inversion centre the molecule is centered. Due to the

low occupancy of these disordered groups, the connectivity has not been completed, as the resulting geometries would not be correct if connected to the remained of the resolved structure. This also results in a slight overoccupancy of oxygen, hydrogen and carbon within the structure, as it is not possible to refine the occupancies of all parts competitively to equal 100%. PLATON CheckCIF calculates that this structure exhibits a large range of non-solvent Ueq's for carbon atoms in the structure. However, despite the assertions of PLATON, this measure has included the disordered and admittedly poorly localised solvent molecules (as can be seen from the moiety formula in the CheckCIF report for this structure, C56.88 H104.21 Cr4 F6 N2.32 O19.70 Zn , rather than the reported C91.903 H165.425 Cr8 F12 O36.761 Zn2, C10 H18 N4, 2.643(C4 H8 O), 2(C0.64 H1.921 N0.32); the solvent molecules have not been counted as separate in the moiety formula calculation). Refinement of the model with the solvent molecules removed suppresses this alert.

8

Crystal of the sample were weakly diffracting. As a result, the data were truncated to the observable limit of 0.95Å resolution, resulting in a lower resolution than desired dataset. PLATON CheckCIF flags low Ueqs for some neighbouring carbon atoms in the "main molecule". This is due to the pivot atoms for the t-butyl moieties of the pivalate groups have a low Ueq compared to their neighbours as the neighbours are methyl groups of the t-butyl moieties and have been modelled with large atomic displacement parameters, indicative of statistical disorder of these groups within the crystal.

The data were also found to be non-merohedrally twinned, with two clear reciprocal lattices observed, albeit only slightly rotated from each other (rotated 0.8211 degrees around [-0.65 -0.03 0.76] reciprocal), clearly from two separate crystals or due to crystal cracking. Treating the data as non-merohedrally twinned eliminated a number of artefacts from the electron density difference map compared to data reduction as a single component.

PLATON CheckCIF also calculates the presence of a large U3/U1 ratio for average U(I,j) tensors. This appears to be due to the disordered toluene solvent molecule and the subsequent similar neighbouring atomic displacement parameter and rigid bond restraints applied these atoms. Unfortunately, these atoms are modelled in a poorly defined region of the electron density map and as a result the restraints cause an alignment of the vibrational direction of the atomic displacement parameters. Due to the resolution of the dataset, further modelling of this disordered toluene could not be applied.

Crystallographic data have been deposited with the CCDC (2214198-2214204).

Table S1. Crystallographic parameters for compounds **2-4**

Identification code	**2**	**3**	**4**
Empirical formula	$C_{85}H_{153}Cr_7F_8N_2O_{32}Zn$ ~~$C_{85}H_{153}Cr_7F_8N_2O_{32}Zn$~~	$C_{93}H_{163}Cr_8F_{11}N_2O_{34}Zn$ ~~$C_{93}H_{163}Cr_8F_{11}N_2O_{34}Zn$~~	$C_{107}H_{196}Cr_9F_{12}NO_{37}Zn$ ~~$C_{107}H_{196}Cr_9F_{12}NO_{37}Zn$~~
Formula weight	2296.46 ~~2296.46~~	2543.61 ~~2543.61~~	2850.01 ~~2849.44~~
Temperature/K	150.00(10) ~~150.00(10)~~	150.00(10) ~~150.00(10)~~	100.00(10) ~~100.00(10)~~
Crystal system	monoclinic ~~monoclinic~~	trigonal ~~trigonal~~	orthorhombic ~~orthorhombic~~
Space group	I2/a ~~I2/a~~	P-3c1 ~~P-3c1~~	$Pna2_1$ ~~$Pna2_1$~~
a/Å	34.9561(7) ~~34.9561(7)~~	36.4236(5) ~~36.4236(5)~~	35.7225(7) ~~35.7225(7)~~
b/Å	16.6364(2) ~~16.6364(2)~~	36.4236(5) ~~36.4236(5)~~	17.1341(3) ~~17.1341(3)~~
c/Å	44.4145(9) ~~44.4145(9)~~	19.1203(3) ~~19.1203(3)~~	23.7329(6) ~~23.7329(6)~~
α/°	90 ~~90~~	90 ~~90~~	90 ~~90~~
β/°	111.673(2) ~~111.673(2)~~	90 ~~90~~	90 ~~90~~
γ/°	90 ~~90~~	120 ~~120~~	90 ~~90~~
Volume/Å^3	24003.0(8) ~~24003.0(8)~~	21968.0(7) ~~21968.0(7)~~	14526.3(5) ~~14526.3(5)~~
Z	8 ~~8~~	6 ~~6~~	4 ~~4~~
ρ_{calc}g/cm^3	1.271 ~~1.271~~	1.154 ~~1.154~~	1.303 ~~1.303~~
μ/mm^{-1}	0.883 ~~0.883~~	5.498 ~~5.498~~	0.889 ~~0.885~~
F(000)	9624.0 ~~9624.0~~	7968.0 ~~7968.0~~	5980.0 ~~5979.0~~
Crystal size/mm^3	0.46 × 0.43 × 0.22 ~~0.46 × 0.43 × 0.22~~	0.398 × 0.174 × 0.145 ~~0.398 × 0.174 × 0.145~~	0.45 × 0.43 × 0.22 ~~0.45 × 0.43 × 0.22~~
Radiation	Mo Kα (λ = 0.71073) ~~Mo Kα (λ = 0.71073)~~	CuKα (λ = 1.54184) ~~CuKα (λ = 1.54184)~~	Mo Kα (λ = 0.71073) ~~Mo Kα (λ = 0.71073)~~
2Θ range for data collection/°	3.38 to 58.284 ~~3.38 to 58.284~~	5.604 to 154.454 ~~5.604 to 154.454~~	3.146 to 61.866 ~~3.146 to 61.866~~
Index ranges	-42 ≤ h ≤ 47, -21 ≤ k ≤ 22, -58 ≤ l ≤ 58 ~~−42 ≤ h ≤ 47, −21 ≤ k ≤ 22, −58 ≤ l ≤ 58~~	-46 ≤ h ≤ 42, -45 ≤ k ≤ 42, -23 ≤ l ≤ 24 ~~−46 ≤ h ≤ 42, −45 ≤ k ≤ 42, −23 ≤ l ≤ 24~~	-44 ≤ h ≤ 47, -23 ≤ k ≤ 20, -31 ≤ l ≤ 29 ~~−44 ≤ h ≤ 47, −23 ≤ k ≤ 20, −31 ≤ l ≤ 29~~
Reflections collected	138780 ~~138780~~	37098 ~~37098~~	186600 ~~186600~~
Independent reflections	28907 [R_{int} = 0.0298, R_{sigma} = 0.0269] ~~28907 [R_{int} = 0.0298, R_{sigma} = 0.0269]~~	37098 [R_{int} = ?, R_{sigma} = 0.0762] ~~37098 [R_{int} = ?, R_{sigma} = 0.0762]~~	38000 [R_{int} = 0.0572, R_{sigma} = 0.0578] ~~38000 [R_{int} = 0.0572, R_{sigma} = 0.0578]~~
Data/restraints/parameters	28907/7801/1677 ~~28907/2362/1616~~	37098/4069/1259 ~~37098/4069/1259~~	38000/10781/2088 ~~38000/1655/1860~~
Goodness-of-fit on F^2	1.054 ~~1.044~~	1.045 ~~1.045~~	1.017 ~~1.011~~
Final R indexes [I>=2σ (I)]	R_1 = 0.0423, wR_2 = 0.0950 ~~R_1 = 0.0431, wR_2 = 0.0987~~	R_1 = 0.1326, wR_2 = 0.2866 ~~R_1 = 0.1326, wR_2 = 0.2866~~	R_1 = 0.0508, wR_2 = 0.0989 ~~R_1 = 0.0564, wR_2 = 0.1226~~
Final R indexes [all data]	R_1 = 0.0573, wR_2 = 0.1003 ~~R_1 = 0.0582, wR_2 = 0.1044~~	R_1 = 0.2110, wR_2 = 0.3416 ~~R_1 = 0.2110, wR_2 = 0.3416~~	R_1 = 0.0785, wR_2 = 0.1072 ~~R_1 = 0.0840, wR_2 = 0.1330~~
Largest diff. peak/hole / e Å^{-3}	0.57/-0.52 ~~0.57/−0.69~~	0.99/-1.18 ~~0.99/−1.18~~	0.45/-0.43 ~~0.73/−0.53~~
Flack parameter			0.027(11) ~~0.026(13)~~

Table S1 continued. Crystallographic parameters for compounds **5-7**

Identification code	**5**	**6**	**7**
Empirical formula	$C_{89.5}H_{158}Cr_6F_8N_4O_{32}Zn_2$ ~~$C_{89.5}H_{158}Cr_6F_8N_4O_{32}Zn_2$~~	$C_{121}H_{203}Cr_8F_{12}N_5O_{36}Zn_2$ ~~$C_{119}H_{200}Cr_8F_{12}N_4O_{36}Zn_2$~~	$C_{113.68}H_{208.26}Cr_8F_{12}N_{4.63}O_{39.38}Zn_2$ ~~$C_{113.64}H_{208.83}Cr_8F_{12}N_{4.78}O_{39.02}Zn_2$~~
Formula weight	2396.93 ~~2396.93~~	3078.61 ~~3037.56~~	3045.00 ~~3041.33~~
Temperature/K	150.0(2) ~~293.15~~	150.00(10) ~~150.00(10)~~	150.00(10) ~~150.00(10)~~
Crystal system	monoclinic ~~monoclinic~~	monoclinic ~~monoclinic~~	orthorhombic ~~orthorhombic~~
Space group	$P2_1/c$ ~~$P2_1/c$~~	C2/c ~~C2/c~~	Cmce ~~Cmce~~
a/Å	25.1079(6) ~~25.1079(6)~~	25.4202(18) ~~25.4202(18)~~	33.2596(4) ~~33.2596(4)~~
b/Å	16.8130(3) ~~16.8130(3)~~	22.0090(8) ~~22.0090(8)~~	20.8631(3) ~~20.8631(3)~~
c/Å	31.3860(8) ~~31.3860(8)~~	29.4754(12) ~~29.4754(12)~~	22.6682(3) ~~22.6682(3)~~
α/°	90 ~~90~~	90 ~~90~~	90 ~~90~~
β/°	111.085(3) ~~111.085(3)~~	99.155(5) ~~99.155(5)~~	90 ~~90~~
γ/°	90 ~~90~~	90 ~~90~~	90 ~~90~~
Volume/Å^3	12362.2(5) ~~12362.2(5)~~	16280.6(14) ~~16280.6(14)~~	15729.4(4) ~~15729.4(4)~~
Z	4 4	4 4	4 4
ρ_{calc}g/cm^3	1.288 ~~1.288~~	1.256 ~~1.239~~	1.286 ~~1.284~~
μ/mm^{-1}	0.966 ~~0.966~~	5.203 ~~5.194~~	5.393 ~~5.391~~
F(000)	5020.0 ~~5020.0~~	6448.0 ~~6360.0~~	6391.0 ~~6385.0~~
Crystal size/mm^3	0.16 × 0.12 × 0.1 ~~0.16 × 0.12 × 0.1~~	0.313 × 0.226 × 0.204 ~~0.313 × 0.226 × 0.204~~	0.335 × 0.228 × 0.113 ~~0.335 × 0.228 × 0.113~~
Radiation	Mo Kα (λ = 0.71073) ~~Mo Kα (λ = 0.71073)~~	Cu Kα (λ = 1.54184) ~~Cu Kα (λ = 1.54184)~~	CuKα (λ = 1.54184) ~~CuKα (λ = 1.54184)~~
2Θ range for data collection/°	2.982 to 58.186 ~~2.982 to 58.186~~	5.34 to 143.438 ~~5.34 to 143.438~~	8.476 to 154.266 ~~8.476 to 154.266~~
Index ranges	-29 ≤ h ≤ 33, -18 ≤ k ≤ 22, -39 ≤ l ≤ 42 ~~−29 ≤ h ≤ 33, −18 ≤ k ≤ 22, −39 ≤ l ≤ 42~~	-31 ≤ h ≤ 29, -26 ≤ k ≤ 26, -36 ≤ l ≤ 31 ~~−31 ≤ h ≤ 29, −26 ≤ k ≤ 26, −36 ≤ l ≤ 31~~	-41 ≤ h ≤ 28, -26 ≤ k ≤ 25, -28 ≤ l ≤ 28 ~~−41 ≤ h ≤ 28, −26 ≤ k ≤ 25, −28 ≤ l ≤ 28~~
Reflections collected	74940 ~~74940~~	69920 ~~69927~~	31103 ~~31103~~
Independent reflections	28605 [R_{int} = 0.0458, R_{sigma} = 0.0732] ~~28605 [R_{int} = 0.0458, R_{sigma} = 0.0732]~~	14630 [R_{int} = 0.0589, R_{sigma} = 0.0396] ~~14632 [R_{int} = 0.0589, R_{sigma} = 0.0396]~~	8250 [R_{int} = 0.0341, R_{sigma} = 0.0302] ~~8250 [R_{int} = 0.0341, R_{sigma} = 0.0302]~~
Data/restraints/parameters	28605/2438/1670 ~~28605/2274/1670~~	14630/5288/1541 ~~14632/5277/1529~~	8250/2215/870 ~~8250/1834/810~~
Goodness-of-fit on F^2	1.016 ~~1.024~~	1.061 ~~1.057~~	1.036 ~~1.035~~
Final R indexes [I>=2σ (I)]	R_1 = 0.0598, wR_2 = 0.1235 ~~R_1 = 0.0631, wR_2 = 0.1353~~	R_1 = 0.0662, wR_2 = 0.2035 ~~R_1 = 0.0726, wR_2 = 0.2215~~	R_1 = 0.0490, wR_2 = 0.1428 ~~R_1 = 0.0632, wR_2 = 0.1895~~
Final R indexes [all data]	R_1 = 0.1068, wR_2 = 0.1389 ~~R_1 = 0.1098, wR_2 = 0.1516~~	R_1 = 0.0848, wR_2 = 0.2178 ~~R_1 = 0.0911, wR_2 = 0.2361~~	R_1 = 0.0651, wR_2 = 0.1562 ~~R_1 = 0.0790, wR_2 = 0.2068~~
Largest diff. peak/hole / e Å^{-3}	0.92/-0.43 ~~0.97/−0.41~~	0.66/-0.82 ~~0.69/−0.84~~	0.41/-0.38 ~~0.91/−0.56~~

Table S1 continued. Crystallographic parameters for compound **8**

Identification code	**8**
Empirical formula	$C_{176.4}H_{308.17}Cr_{12}F_{16}N_2O_{52}Zn_2$ ~~$C_{176.4}H_{308.17}Cr_{12}F_{16}N_2O_{52}Zn_2$~~
Formula weight	4347.94 ~~4347.94~~
Temperature/K	150.02(10) ~~150.02(10)~~
Crystal system	triclinic ~~triclinic~~
Space group	P-1 ~~P-1~~
a/Å	13.5561(7) ~~13.5561(7)~~
b/Å	20.1031(12) ~~20.1031(12)~~
c/Å	21.8278(12) ~~21.8278(12)~~
α/°	94.884(5) ~~94.884(5)~~
β/°	92.430(5) ~~92.430(5)~~
γ/°	109.377(5) ~~109.377(5)~~
Volume/Å^3	5575.4(6) ~~5575.4(6)~~
Z	1 ~~1~~
ρ_{calc}g/cm^3	1.295 ~~1.295~~
μ/mm^{-1}	0.851 ~~0.851~~
F(000)	2289.0 ~~2289.0~~
Crystal size/mm^3	0.296 × 0.161 × 0.102 ~~0.296 × 0.161 × 0.102~~
Radiation	Mo Kα (λ = 0.71073) ~~Mo Kα (λ = 0.71073)~~
2Θ range for data collection/°	5.488 to 43.934 ~~5.488 to 43.934~~
Index ranges	$-13 \leq h \leq 14$, $-16 \leq k \leq 21$, $-22 \leq l \leq 22$ ~~$-13 \leq h \leq 14$, $-16 \leq k \leq 21$, $-22 \leq l \leq 22$~~
Reflections collected	13733 ~~13733~~
Independent reflections	13733 [R_{int} = 0.0913, R_{sigma} = 0.3426] ~~13733 [R_{int} = 0.0913, R_{sigma} = 0.3426]~~
Data/restraints/parameters	13733/5044/1486 ~~13733/5044/1486~~
Goodness-of-fit on F^2	0.880 ~~0.880~~
Final R indexes [I>=2σ (I)]	R_1 = 0.0580, wR_2 = 0.1179 ~~R_1 = 0.0580, wR_2 = 0.1179~~
Final R indexes [all data]	R_1 = 0.1315, wR_2 = 0.1307 ~~R_1 = 0.1315, wR_2 = 0.1307~~
Largest diff. peak/hole / e Å^{-3}	0.70/-0.51 ~~0.70/-0.51~~

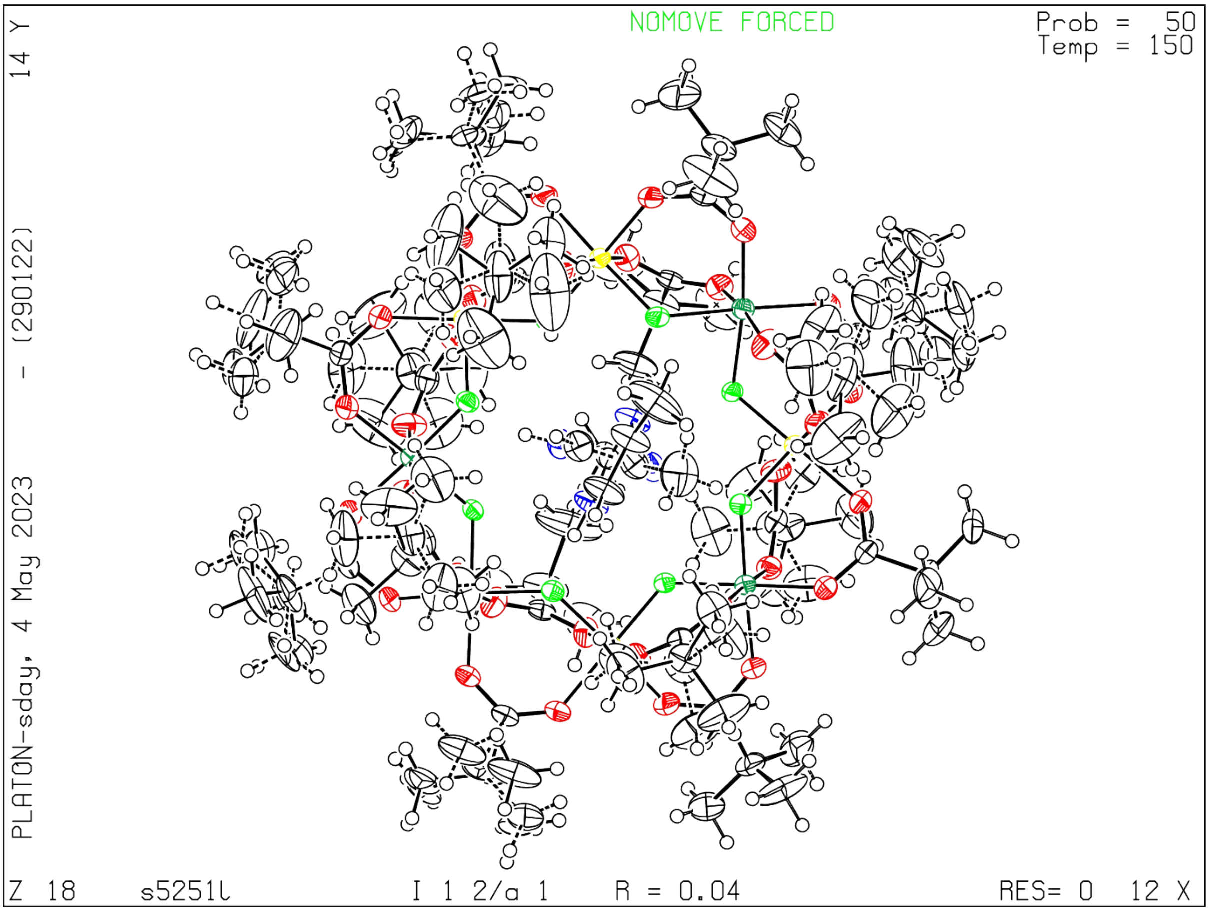

Figure S1: Ortep plot of **2** generated using PLUTON. White = carbon, red = oxygen, blue = nitrogen, light green = fluorine, dark green = chromium, yellow = zinc. Hydrogens included as small white circles. Disorder has been included in the plot.

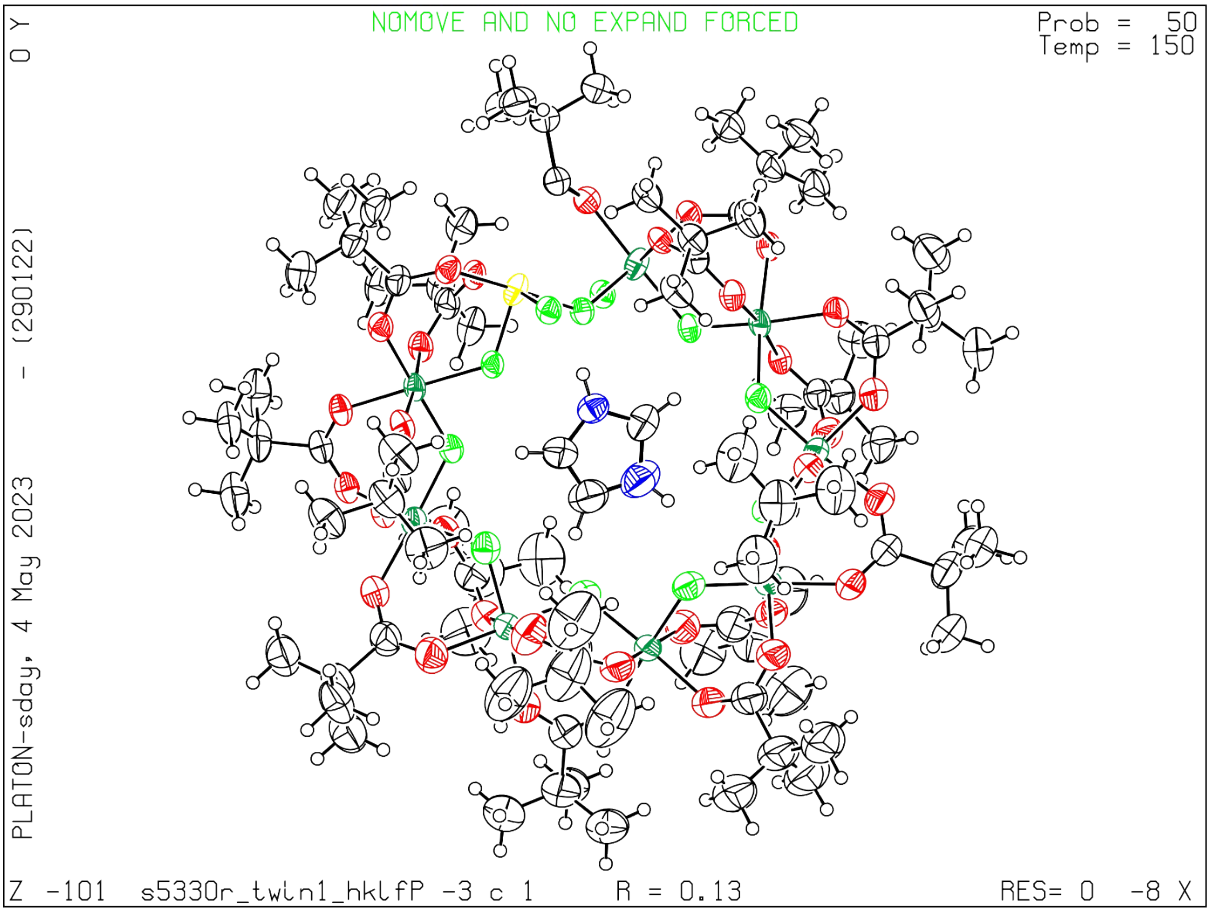

Figure S2: Ortep plot of **3** generated using PLUTON. White = carbon, red = oxygen, blue = nitrogen, light green = fluorine, dark green = chromium, yellow = zinc. Hydrogens included as small white circles. Disorder has been included in the plot. Note: some bonds are missing due to the way the model was constructed; PLUTON cannot detect added bonds between parts.

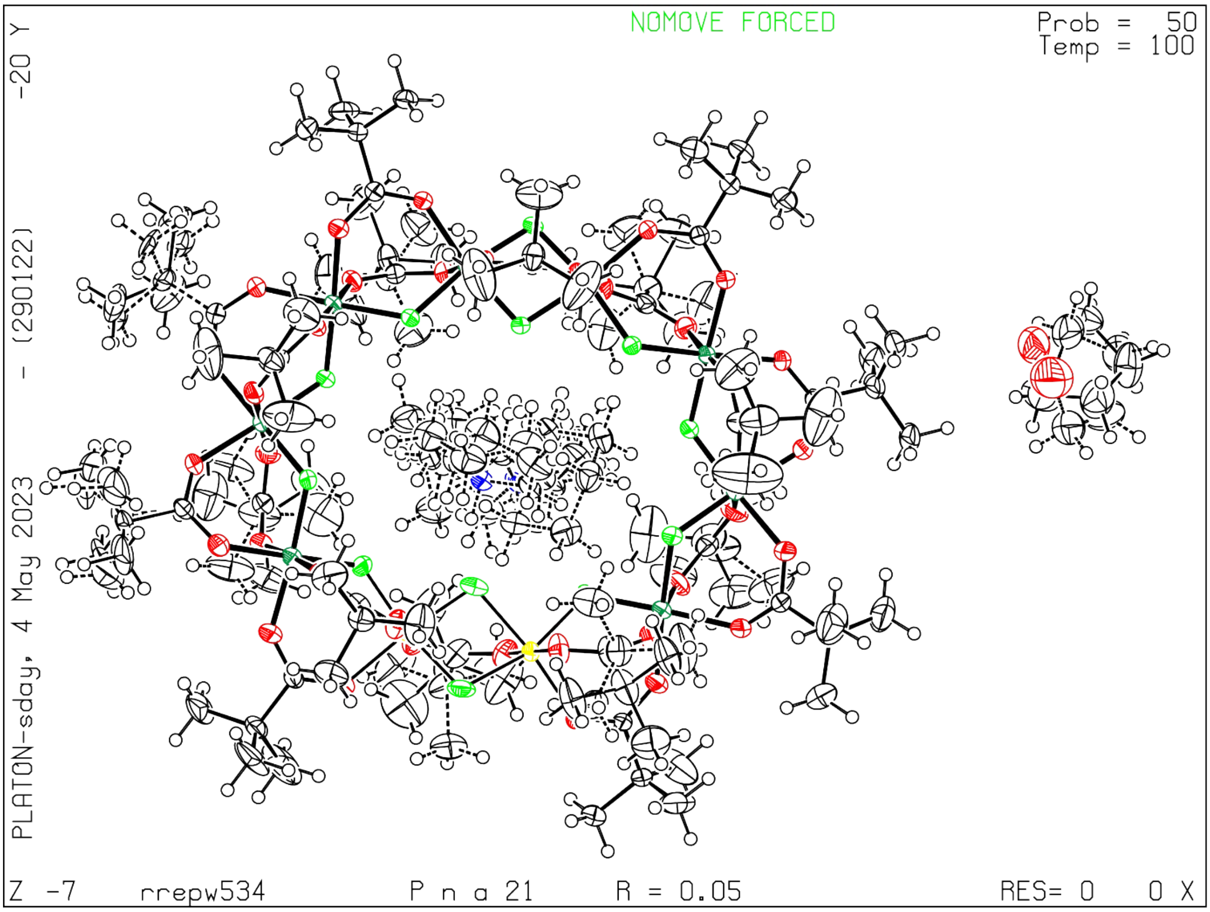

Figure S3: Ortep plot of **4** generated using PLUTON. White = carbon, red = oxygen, blue = nitrogen, light green = fluorine, dark green = chromium, yellow = zinc. Hydrogens included as small white circles. Disorder has been included in the plot.

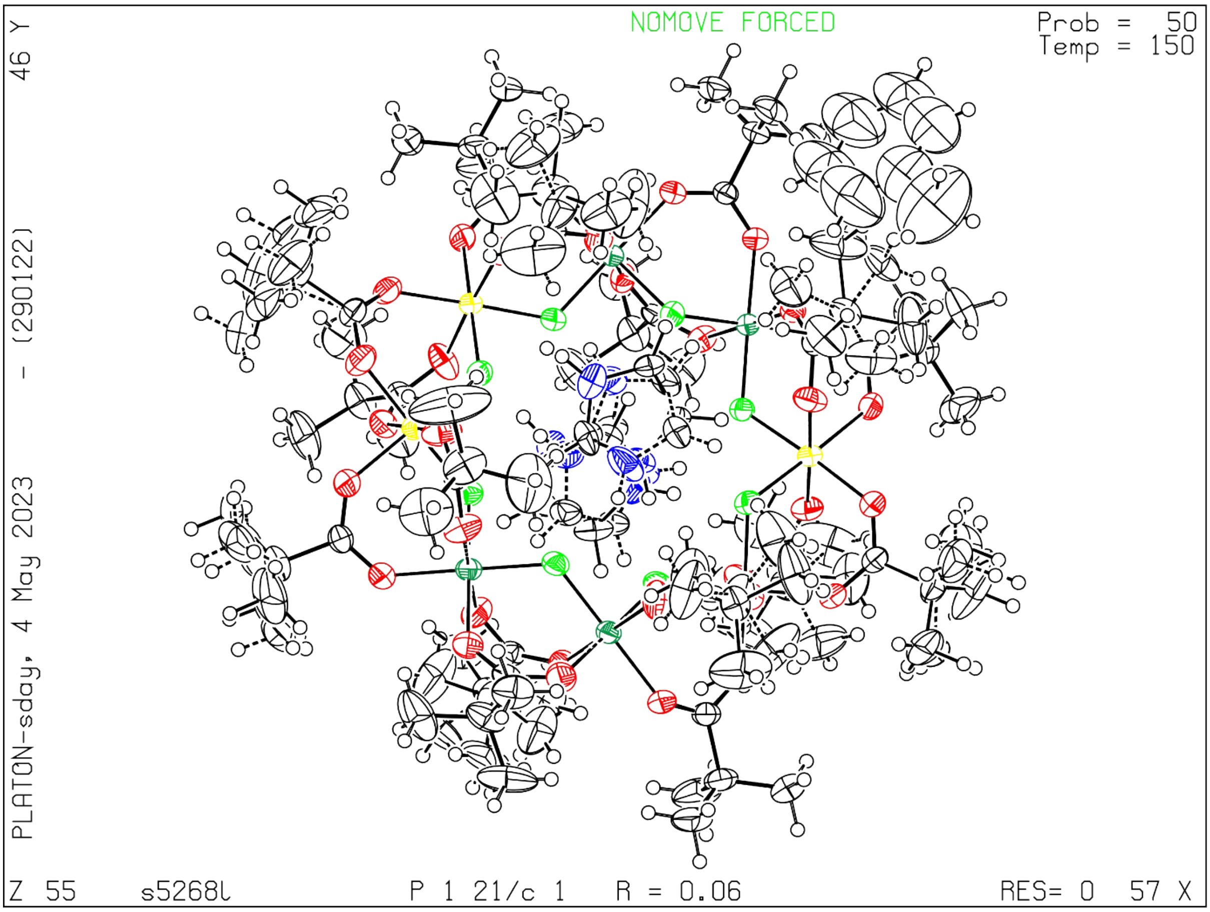

Figure S4: Ortep plot of **5** generated using PLUTON. White = carbon, red = oxygen, blue = nitrogen, light green = fluorine, dark green = chromium, yellow = zinc. Hydrogens included as small white circles. Disorder has been included in the plot.

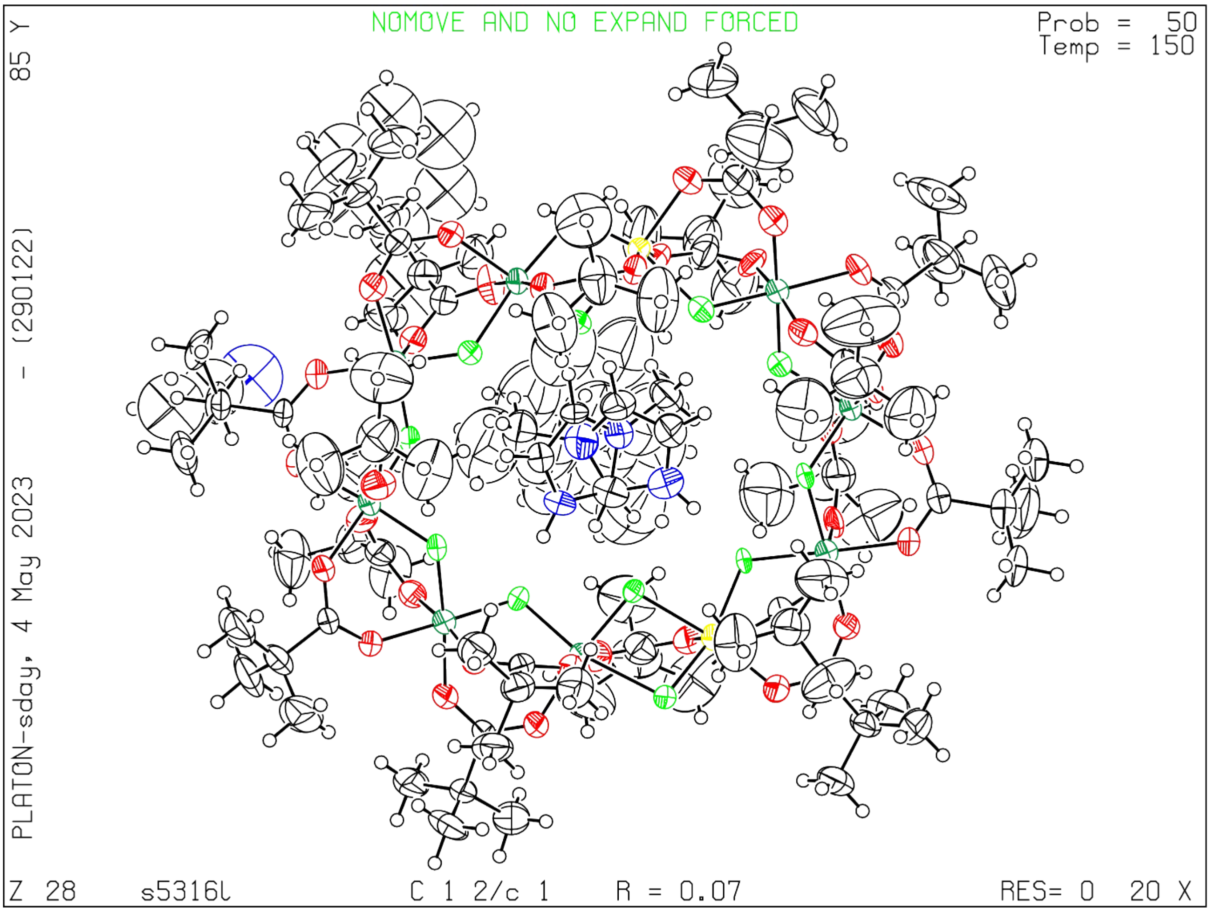

Figure S5: Ortep plot of **6** generated using PLUTON. White = carbon, red = oxygen, blue = nitrogen, light green = fluorine, dark green = chromium, yellow = zinc. Hydrogens included as small white circles. Disorder has been included in the plot.

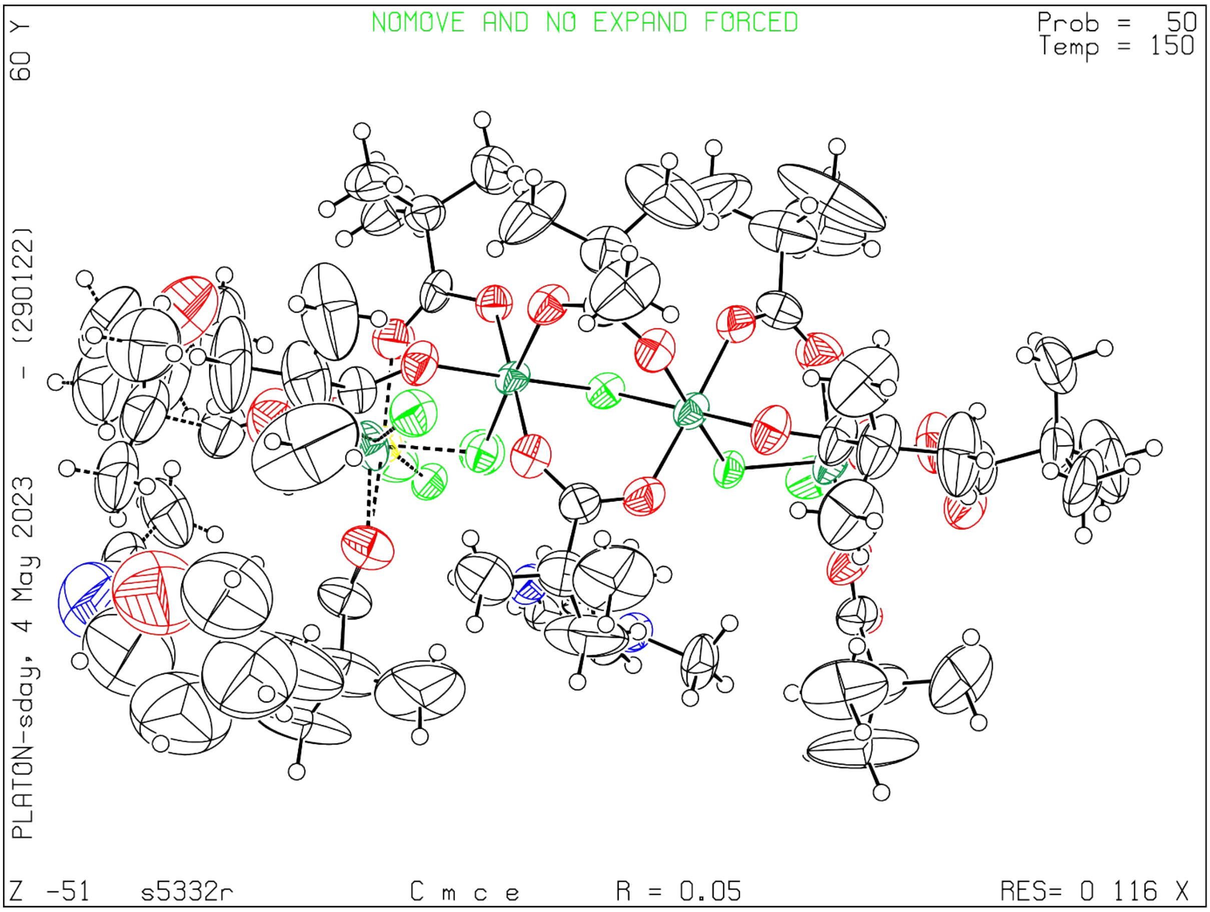

Figure S6: Ortep plot of **7** generated using PLUTON. White = carbon, red = oxygen, blue = nitrogen, light green = fluorine, dark green = chromium, yellow = zinc. Hydrogens included as small white circles. Disorder has been included in the plot. Note: Model displayed as half molecule due to the way the model was constructed; PLUTON cannot detect added bonds between parts.

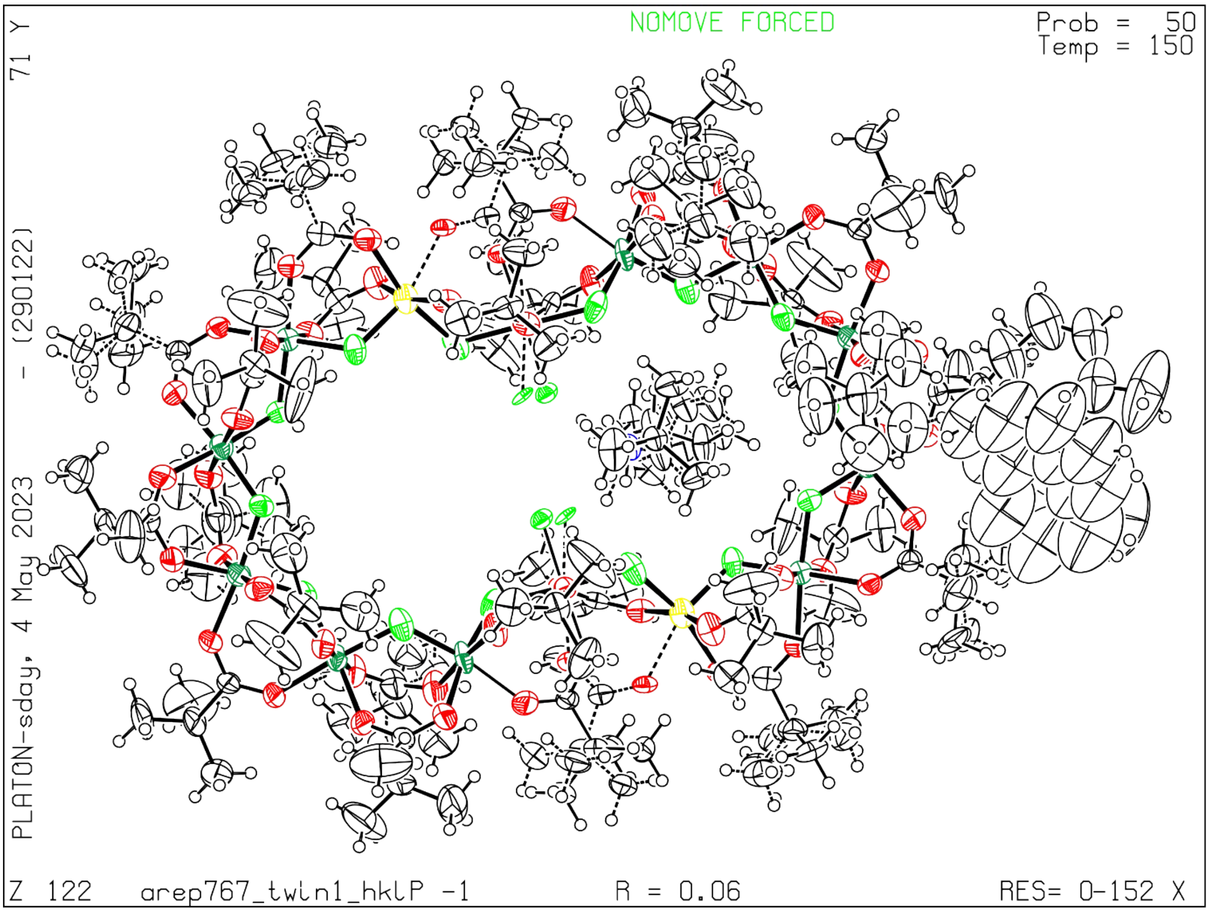

Figure S7: Ortep plot of **8** generated using PLUTON. White = carbon, red = oxygen, blue = nitrogen, light green = fluorine, dark green = chromium, yellow = zinc. Hydrogens included as small white circles. Disorder has been included in the plot.

S3. Magnetic Data

All magnetic measurements were performed using a Quantum Design MPMS-XL7 SQUID magnetometer equipped with a 7 T magnet.

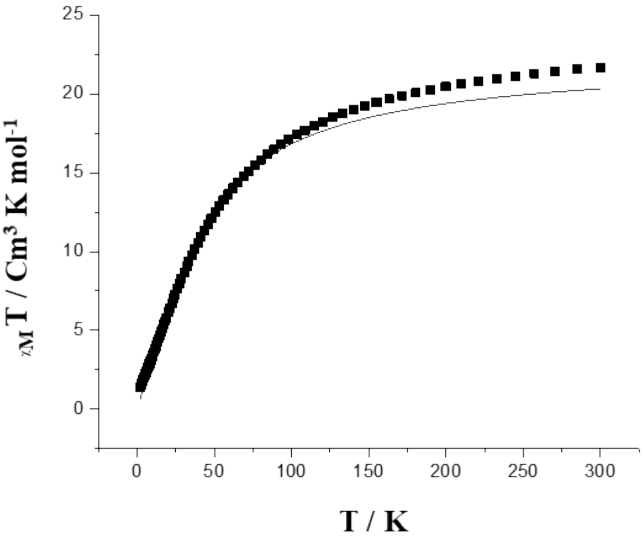

Figure S1: Temperature-dependent χ_MT of **8** in the temperature range 2 to 300 K, (measured at 1000 Oe); calculation for $J = -5.2$ cm^{-1}, $g = 1.99$, the experimental data (■) and simulation (—).

S4. EPR Spectroscopy

Experimental details. All data were recorded on powder samples. Q-band data were taken on a Bruker E500 spectrometer equipped with an ER 5106 QT (Q-band, *ca*. 34 GHz) resonator. K-band data were collected on a Bruker E580 spectrometer equipped with an ER 6706KT (K-band, (*ca*. 24 GHz) resonator.

Coupled spin states were determined for chromium ion chains of length n by generating the Hamiltonian:

$$\hat{H} = \sum_{k=1}^{n} gB_0\hat{S}_k - \sum_{k=1}^{n-1} 2J\hat{S}_{k-1}\hat{S}_k$$

Where B_0 is the applied magnetic field vector, $\hat{S}_k$ are the spin operators for the kth spin in the Cartesian basis set, and assuming $g = 1.96$ and $J = -5.89$ cm^{-1}, taken from Reference 25 in the main text. The field B_0 was stepped from 0.01 to 0.05 G in the z-direction, and the Hamiltonian was diagonalised to obtain the energy levels of the spin states, which due to the simplified Hamiltonian could be fitted with a straight line $E = E_{coup} + m_s hgB$ to determine the spin projection from the gradient, and the energy in the absence of an applied field as the intercept.

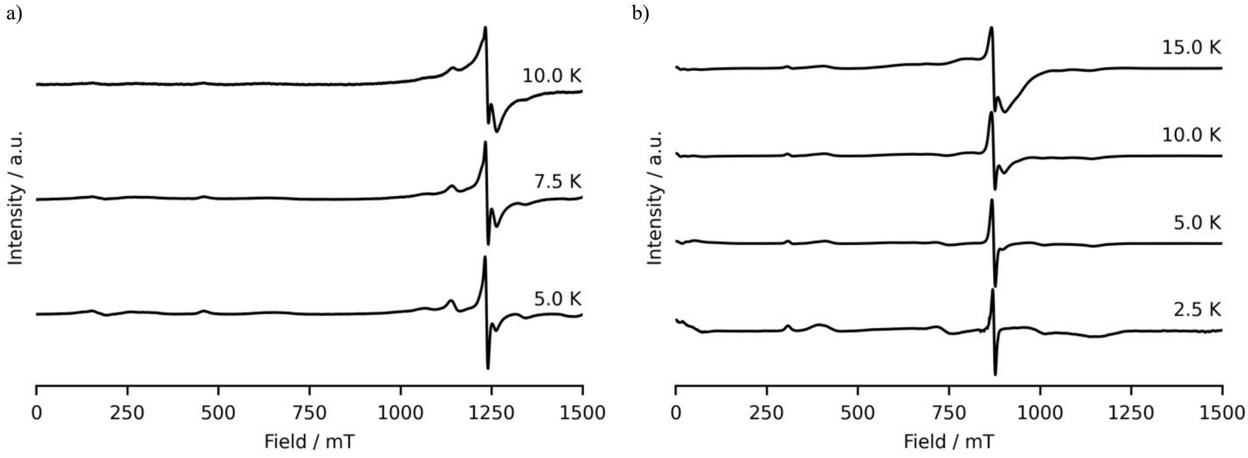

Figure S2: Variable temperature EPR spectra of polycrystalline **2** at a) Q- and b) K-band (34 GHz and 24 GHz, respectively).

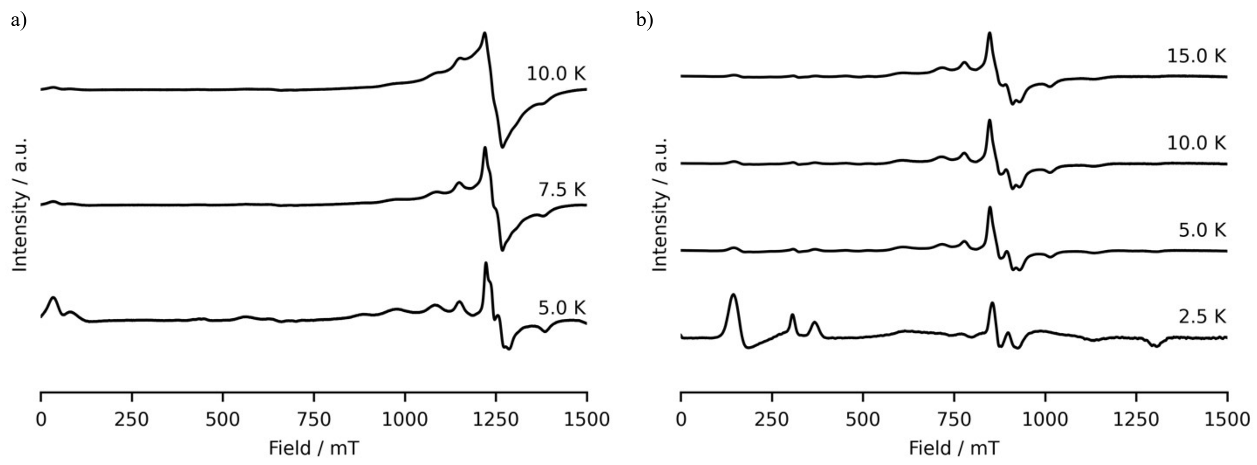

Figure S3: Variable temperature EPR spectra of polycrystalline **4** at a) Q- and b) K-band (34 GHz and 24 GHz, respectively).

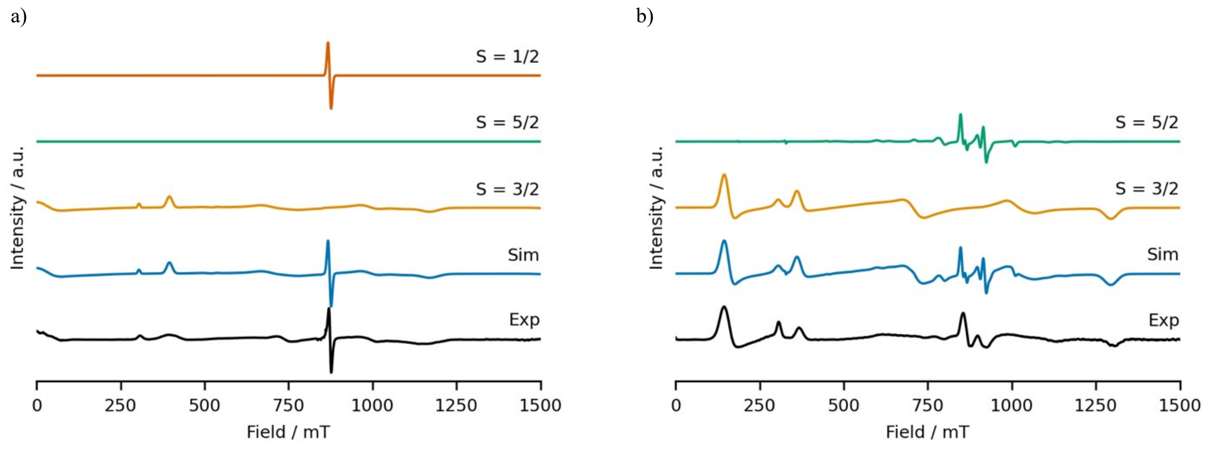

Figure S4: The EPR spectra (black) and simulations (blue) of polycrystalline samples of a) **2** and b) $\mathbf{4_{Zn}}$ at K-band (24 GHz) at 2.5 K. The individual components of the simulations are shown stacked above the complete simulation, and the parameters used for these simulations are listed in Table 1 of the main text.

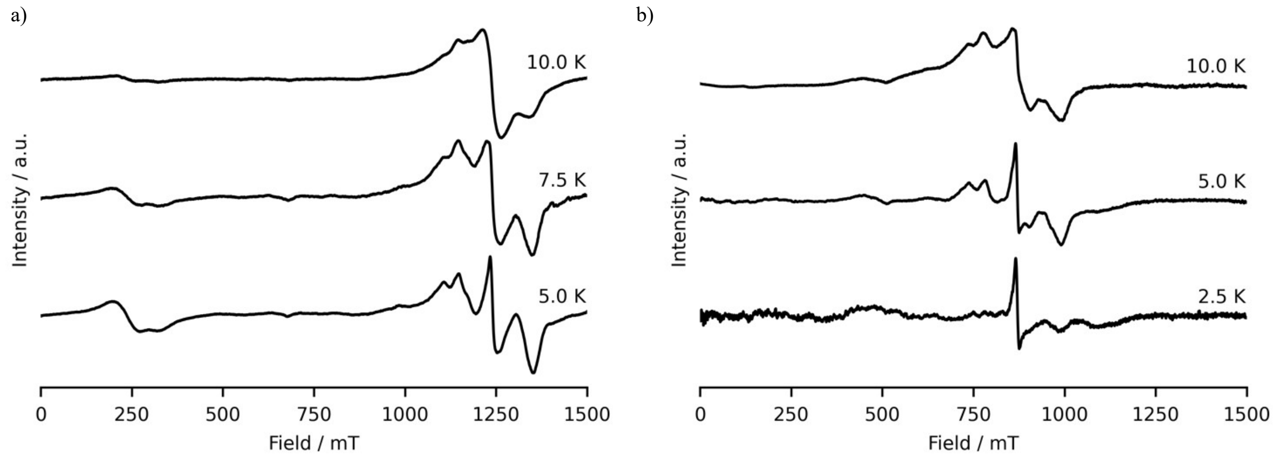

Figure S5: Variable temperature EPR spectra of polycrystalline **8** at a) Q- and b) K-band (34 GHz and 24 GHz, respectively).

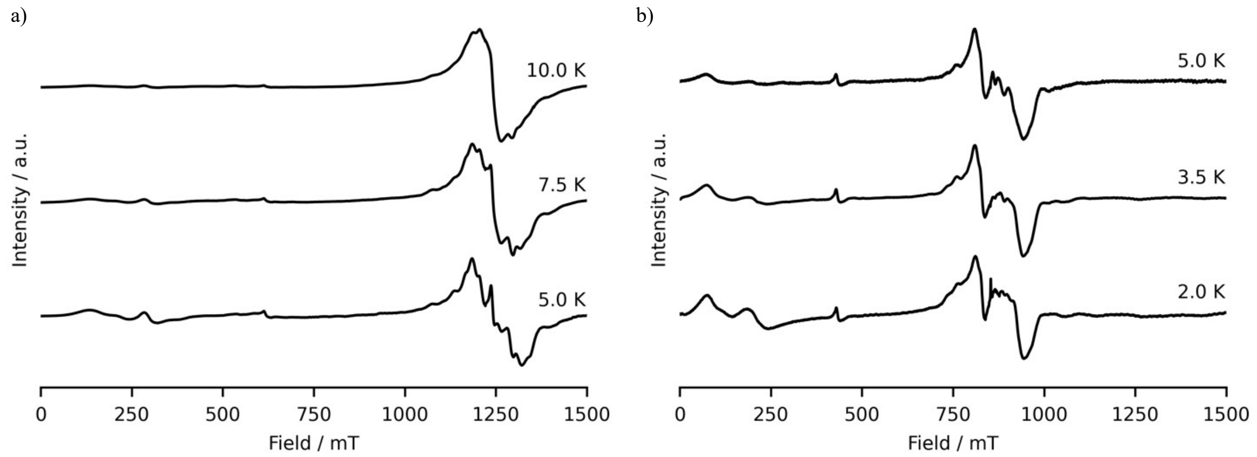

Figure S6: Variable temperature EPR spectra of polycrystalline **3** at a) Q- and b) K-band (34 GHz and 24 GHz, respectively).

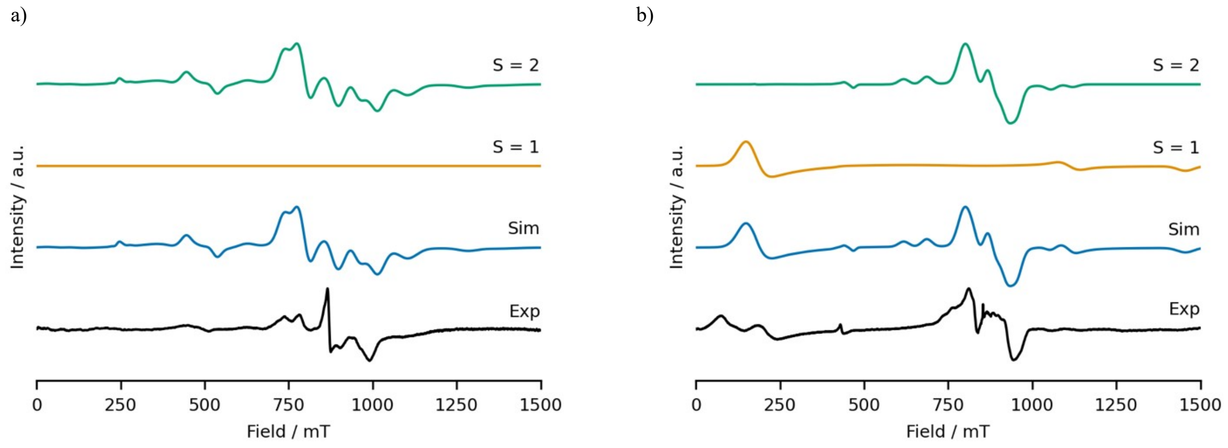

Figure S7: The EPR spectra (black) and simulations (blue) of polycrystalline samples of a) **8** at 5 K and b) **3** at K-band (24 GHz) at 2 K. The individual components of the simulations are shown stacked above the complete simulation, and the parameters used for these simulations are listed in Table 1 of the main text.

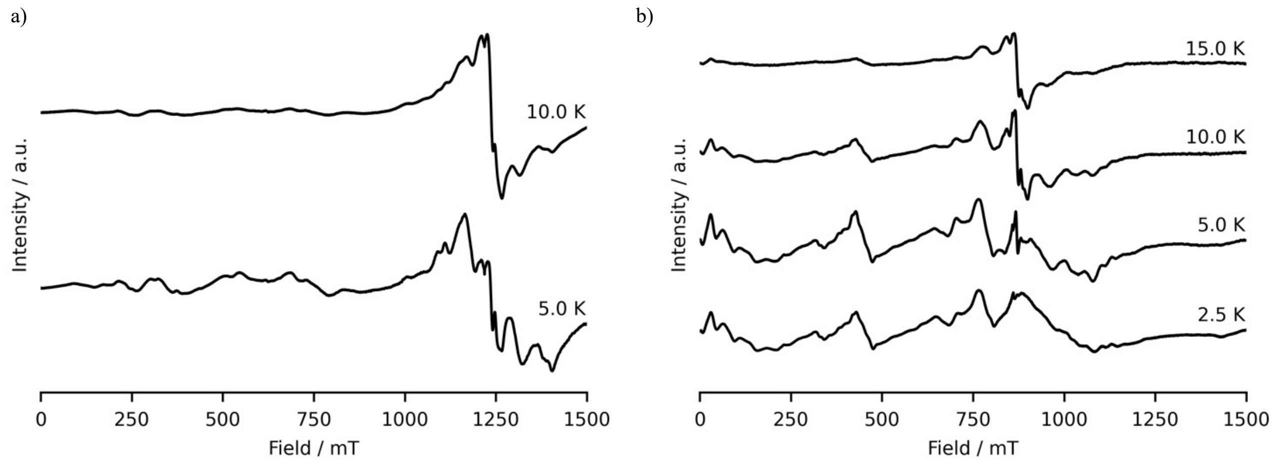

Figure S8: Variable temperature EPR spectra of polycrystalline **5** at a) Q- and b) K-band (34 GHz and 24 GHz, respectively).

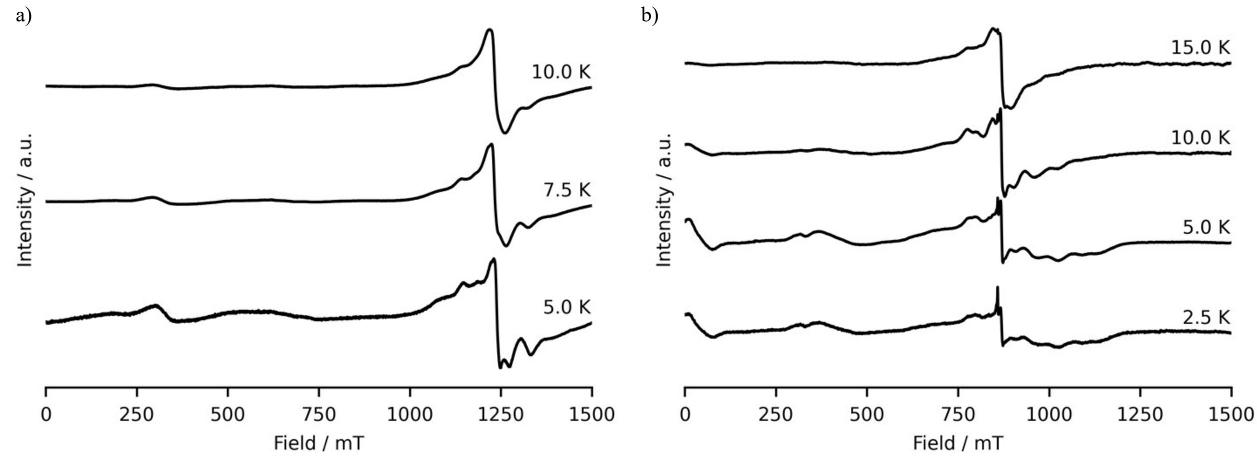

Figure S9: Variable temperature EPR spectra of polycrystalline **6** at a) Q- and b) K-band (34 GHz and 24 GHz, respectively).

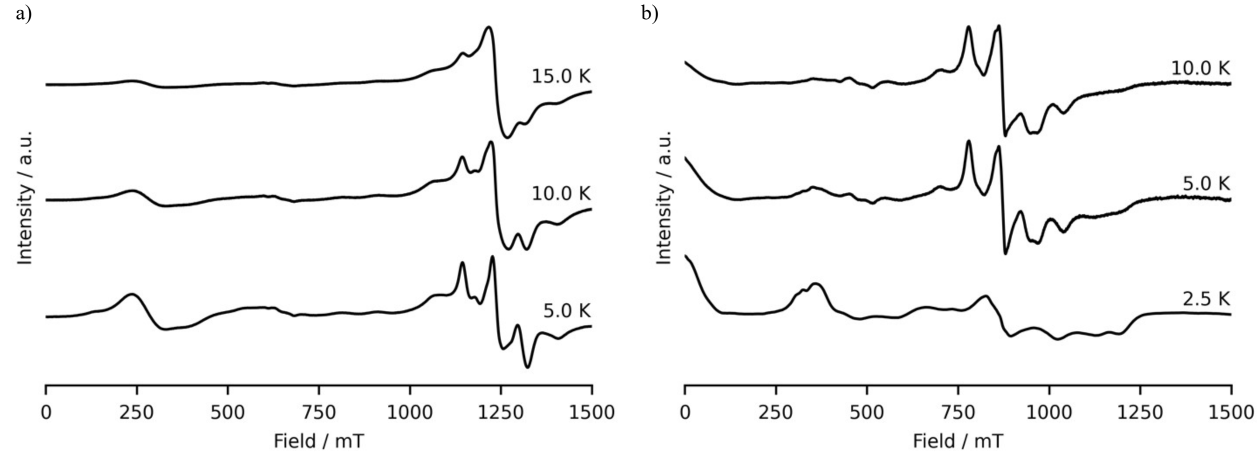

Figure S10: Variable temperature EPR spectra of polycrystalline **7** at a) Q- and b) K-band (34 GHz and 24 GHz, respectively).

S5. References

1- Rigaku Oxford Diffraction, CrysAlisPro Software system, version 42, Rigaku Corporation, Oxford, UK, **2017**

2 - G. M. Sheldrick, *Acta. Cryst.*, **2015**, *A71*, 3-8.

3 - O. V. Dolomanov, L. J. Bourhis, R. J. Gildea, J. A. K. Howard and H. Puschmann, J. *Appl. Cryst.*, **2009**, *42*, 339-341
